# Tamoxifen inhibits cell proliferation by impaired glucose metabolism in gallbladder cancer

**DOI:** 10.1111/jcmm.14851

**Published:** 2019-11-28

**Authors:** Shuai Huang, Hui Wang, Wei Chen, Ming Zhan, Sunwang Xu, Xince Huang, Ruirong Lin, Hui Shen, Jian Wang

**Affiliations:** ^1^ Department of Biliary‐Pancreatic Surgery Renji Hospital School of Medicine Shanghai Jiao Tong University Shanghai China

**Keywords:** AMPK, chemoresistance, gallbladder cancer, glycolysis, ROS, tamoxifen

## Abstract

Gallbladder cancer (GBC) is the leading malignancy of biliary system showing refractory chemoresistance to current first‐line drugs. Growing epidemiological evidences have established that the incidence of GBC exhibits significant gender predominance with females two‐threefold higher than males, suggesting oestrogen/oestrogen receptors (ERs) signalling might be a critical driver of tumorigenesis in gallbladder. This study aims to evaluate the antitumour activity of tamoxifen (TAM), a major agent of hormonal therapy for breast cancer, in preclinical GBC model. Quantitative real‐time PCR was used to investigate mRNA levels. Protein expression was measured by immunohistochemistry and Western blot. Glycolytic levels were measured by glucose consumption and lactic acid measurement. The antitumour activity of TAM alone or with cisplatin was examined with CCK8 assay, colony formation, flow cytometry and in vivo models. The results revealed that ERɑ expression was higher in GBC tissues and predicted poor clinical outcomes. TAM was showed effective against a variety of GBC cell lines. Mechanical investigations revealed that TAM enabled potent reactive oxygen species (ROS) production by reduced nuclear factor Nrf2 expression and its target genes, leading to the activation of AMPK, which subsequently induced impaired glycolysis and survival advantages. Notably, TAM was demonstrated to sensitize GBC cells to cisplatin (CDDP) both in vitro and in vivo. In agreement with these findings, elimination of oestrogens by ovariectomy in nude mice prevented CDDP resistance. In summary, these results provide basis for TAM treatment for GBC and shed novel light on the potential application of endocrine therapy for patients with GBC.

## 1 INTRODUCTION

Gallbladder cancer (GBC) is the most common aggressive tumour of the biliary system. Latest epidemiological studies demonstrated that global incidence of gallbladder cancer showed an apparent upward trend, especially in countries from Southeast Asia and South America.^1^, ^2^ Despite the availability of radical resection, most patients are diagnosed with GBC at advanced stages and become surgically unresectable due to the insidious onset and lack of typical clinical manifestations.^3^ Cisplatin‐based chemotherapeutic regimen is regarded as first‐line treatment for patients with advanced disease, only limited response and survival benefits were observed, owing to the extensive chemoresistance of GBC.^4^, ^5^ The striking prevalence of chemoresistance associated with worse prognosis implies the need for new drugs that display a promising efficacy and potentiate the cytotoxic effects of CDDP. Therefore, identification of new roles (indication) of old drugs (clinical drugs) is thought to be of great importance for rapid drug development and would provide promising potential for clinical use.^6^

Emerging studies have indicated gallbladder cancer showed obvious gender bias. An estimated 2‐3 times higher incidence was found in females than males. In addition, the incidence of gallbladder cancer can be increased by endogenousoestrogen. Use of contraceptive pills, multiple births, the birth age of the last foetus, oral hormonal replacement therapy or obesity is associated with increased risk of gallbladder cancer, indicating the important effect of oestrogens in carcinogenesis.^7^, ^8^, ^9^, ^10^

Tamoxifen is a typical anti‐oestrogen drug which has achieved quite satisfactory results in breast cancer treatment and also shows good results in a variety of tumour studies.^11^ Over the past 20 years, there have been more than 25 clinical studies reported that high doses of TAM clinical trials, including melanoma, glioma, have achieved positive results.^12^, ^13^ It is generally believed that TAM plays a major role in the competitive binding and inhibition of ER,^14^, ^15^ while some other studies claimed different mechanism, such as inhibiting protein kinase C (PKC).^16^, ^17^

Based on the known background, we designed this study to explore the therapeutic effects of TAM on GBC and its possible mechanisms. Data here showed TAM was capable of a significant inhibiting effect on the proliferation of gallbladder cancer. It was further revealed that GBC cells receiving TAM decreased Nrf2 expression and its target genes including NQO1 and HO‐1, resulting in elevated ROS production and activation of AMPK pathway. Consequently, activation of AMPK in GBC cells impaired glycolysis and initiated pro‐apoptotic programme. This study also indicated that combination of TAM and cisplatin showed a significantly synergetic effect in GBC cells and nude mice. Combined with the observation that ovariectomized mice showed reduced tumour growth and CDDP resistance, we proposed here that CDDP plus endocrine therapy might represent a potential therapeutic option for improved therapeutic effect and survival benefits of GBC patients.

## 2 MATERIALS AND METHODS

### 2.1 Patients and tissue samples

Clinical information of 250 GBC patients without anticancer therapies before was collected in Renji Hospital (January 2006‐December 2015). Tissue samples of 123 patients fixed by formalin and embedded with paraffin were obtained according to the following criteria. Inclusion criteria: (a) Patients received ultrasound and computed tomography scans prior to surgery. (b) Tissue samples were confirmed by histological proof. (c) Tissue samples and follow‐up information were available. Exclusion criteria: Patients who received preoperative chemotherapy, radiotherapy or other anticancer therapies. About 6 GBC patients were recruited for RNA‐Seq and indicated high expression of ERαin tumour tissue. Twenty‐one pairs of GBC samples and matched non‐cancerous tissues were obtained from 123 patients above. Frozen fresh samples were routinely stored in liquid nitrogen. Two certified pathologists from the Department of Pathology evaluated IHC samples. Study work about involving patient samples was approved by the Ethical Committee of Renji Hospital, Shanghai Jiao Tong University School of Medicine. All patients were well informed, and informed consent was obtained before study. All related experiments were conformed to the approved regulations and guidelines.

### 2.2 Cell culture and transfection

Several human cell lines were used in our study. GBC‐SD, RBE, Patu8988, AsPC1 and HIBEpiC were obtained from the Cell Bank of Type Culture Collection of Chinese Academy of Sciences (Shanghai, China). NOZ was provided by the Health Science Research Resources Bank (Osaka, Japan). QBC‐939 and SGC‐996 were obtained by the Academy of Life Sciences, Tong Ji University (Shanghai, China). GBC‐SD, Patu8988, AsPC1 and HIBEpiC were maintained in DMEM, RBE, SGC‐996 and QBC‐939 were maintained in RPMI‐1640, and NOZ was cultured in William's E medium (Gibco, NY, USA), containing 10% foetal bovine serum (FBS) and antibiotics (Gibco, NY, USA). Phenol red‐free DMEM and Charcoal Stripped Fetal Bovine Serum (Gibco, NY, USA) were used in E2 treatment experiments. Cells were maintained in controlled humidified atmosphere with 5% CO2 under 37°C. Tamoxifen, compound C, DCFDA, NAC and 17β‐estradiol (E2) were purchased from Medchem Express (MCE, USA). For inhibition of AMPK or ERɑ, cells with 65%‐70% confluence were transfected with shRNAs (AMPK: CAGGCCCAGAGGTAGATAT and AGAGAAATTCAGAACCTCA; ERɑ: GCCCTACTACCTGGAGAACGA and CTACAGGCCAAATTCAGATAA) or corresponding controls (GenePharma, Shanghai, China) by Lipofectamine 2000 (Invitrogen). Experiments were performed in accordance to the manufacturer's instructions. After 48 hours, the cells were collected and applied to subsequent experiments.

### 2.3 Glucose consumption measurement

Cells were planted in the six‐well plates and cultured for 24 hours. Then culture media were replaced by 3 mL fresh media with multiple treatments. After certain period, the supernatant was collected and measured for glucose concentration by glucose essay kit (Rsbio). The cells left were counted to standardize glucose consumption levels to nmol min^−1^ per 10^6^ cells.

### 2.4 Lactic acid measurement

The supernatant of cells was collected and measured for lactic acid by colorimetric method. Experiments followed manufacturer's instructions of Lactic Acid assay kit (Nanjing Jiancheng bioengineering Institute, Nanjing, China). The cells left were counted to standardize lactic acid release levels to nmol min^−1^ per 10^6^ cells.

### 2.5 Colony formation

In our colony formation study, suspended cells were plated and cultured in 24‐well plates with a density of 100 per well for 24 h. After drug treatment, GBC cells were maintained in the plate for 14 days. The cell colonies were fixed by 4% paraformaldehyde for 15 minutes and stained with 0.1% crystal violet.

### 2.6 Quantitative real‐time PCR analysis

Total RNA of cultured cells or snap‐frozen tissues was isolated with TRI reagent (Sigma). RNA concentration and purity were measured with NanoDrop ND‐8000 (Thermo Fisher Scientific), and Reverse Transcriptase M‐MLV kit (Invitrogen) was used for cDNAs synthesis. SYBR Premix Ex Taq (Takara) was used to determine expression levels of mRNA by ViiATM 7 Real‐Time PCR System (Applied Biosystems) with 2^−ΔΔCT^ method. The primers were obtained from Sangon Biotech (Shanghai, China) and the sequences are listed in Table S1.

### 2.7 Cytotoxicity, cell apoptosis and cell proliferation assays

Cells were planted in 96‐well plates (5 × 103 cells/well) and maintained overnight. Prepared GBC cells were treated with cisplatin or tamoxifen at multiple concentrations for 48 h. Cell Counting Kit‐8 (Dojindo Laboratories, Japan) was using to measure cell viability every 24 hours in accordance to the manufacturer's instructions. Synergy 2 (BioTek) plate reader was used to measure absorbance at 450 nm. CCK8 assay also could analyse cell proliferation after GBC cells were planted into a 96‐well plates (2 × 10^3^ cells/well) and maintained for 96 h. Annexin V/PI Apoptosis Detection Kit (BD Biosciences) was used to analyse apoptosis of GBC cell lines. Cells were planted in six‐well plates and cultured to about 60% confluence, followed by treatment of cisplatin or tamoxifen for 48 h. Cells were collected and incubated with Annexin V/PI for 15 minutes in dark environment and measured by fluorescence‐activated cell sorting (FACS) analysis.

### 2.8 Western blot analysis

RIPA buffer with proteinase inhibitor cocktail was used to isolated protein from NOZ, GBC‐SD and SGC‐996 cells. BCA assay was used to measure protein concentration. About 10% sodium dodecyl sulphate‐polyacrylamide gel for electrophoresis (SDS‐PAGE) was applied for protein separation followed by transmembrane with PVDF membranes (Millipore). 5% skimmed milk in TBST blocked membranes for 1 hour at room temperature, and primary antibodies incubation were carried out overnight at 4°C. After TBST washing for three times, secondary antibody incubation was applied at room temperature for 2 hours. After TBST washing, chemiluminescence HRP substrate kit (Millipore) was used to detected target protein. The primary antibodies used were as follows: NRF2 (R&D), AMPK, p‐AMPK (Thr172), Cleaved PARP, mTOR, p‐mTOR, LC3 (CST), ERα (Santa Cruz) and ERβ, β ‐actin (Sigma).

### 2.9 Immunohistochemistry

About 10% buffered formalin‐fixed and paraffin‐embedded specimens were cut into 4‐μm thick sections. Standard immunohistochemistry method was used with antibodies staining of Ki67 (1:500, CST). DAB systems detected positive staining cells and haematoxylin was applied for counterstain. Two pathologists knowing nothing about study design assessed final results independently. The staining scores were based on the intensity and proportion of the positive staining. The specimens were classified into followed groups by staining intensity (0 as negative, 1 as weak, 2 as moderate and 3 as strong).

### 2.10 In vivo studies

Animal experiments were strictly performed in according to the guidelines of the Animal Care and Use Committee of Shanghai Jiao Tong University and approved by IACUC committee of Shanghai Jiao Tong University. 1 × 10^6^ GBC‐SD cells in 50 μL medium were transplanted subcutaneously into 6‐week‐old male nude mice. Nude mice were separated into followed groups (group 1, oil + saline; group 2, tamoxifen + saline; group 3, cisplatin + oil; group 4, cisplatin + tamoxifen; n = 8/group; and oil, peanut oil). Two weeks later, the tumour‐bearing mice were treated with CDDP (2 mg/kg) every week, tamoxifen (30 mg/kg) every other day or corresponding dissolvent by intraperitoneal injection. Tamoxifen (20 mg) was dissolved in 1 mL peanut oil. After 34th days, all mice were sacrificed and the tumours were sliced up for IHC and H&E staining. Tumour volumes were measured once every 4 days and calculated by the followed equation: Volume = (Length × Width^2^)/2. To determine systemic toxicity, hearts, livers and kidneys of the mice were histologically examined.

### 2.11 Studies of the ovariectomized mouse model

To induce anaesthesia, 6‐week‐old female mice were injected with 1% pentobarbital sodium (0.06 mL/10 g). Incision position was chosen at 0.4 cm from the intersection of the extension from hind legs and the midline of back. After local disinfection, 2 cm incision was made and fat pad was pulled out. Then, the ovary and fallopian tube were exposed. We ligated fallopian tube and removed the ovary carefully. Lastly, each abdominal wall layer was closed. The control group was operated similarly, and ovaries were intact. Ten days after operation, GBC‐SD cells were transplanted subcutaneously into mice and treatment was followed as plan.

### 2.12 Statistics

Data were shown as mean values ± SEM. Unpaired two‐tailed Student's *t* test was used for two‐group comparisons. Survival probabilities were analysed by Kaplan‐Meier method and log‐rank test. Cox proportional hazard regression model was used for univariate and multivariate analysis. Pearson's *χ* ^2^ test was used for analysis of clinical data. Four replicates were applied in each experiment. SPSS 17.0 was used for statistical analysis. This study considered *P* < .05 statistically significant.

## 3 RESULTS

### 3.1 Altered ERɑ signalling is closely associated with gallbladder carcinogenesis, poor prognosis and a therapeutic target

**Figure 1:**
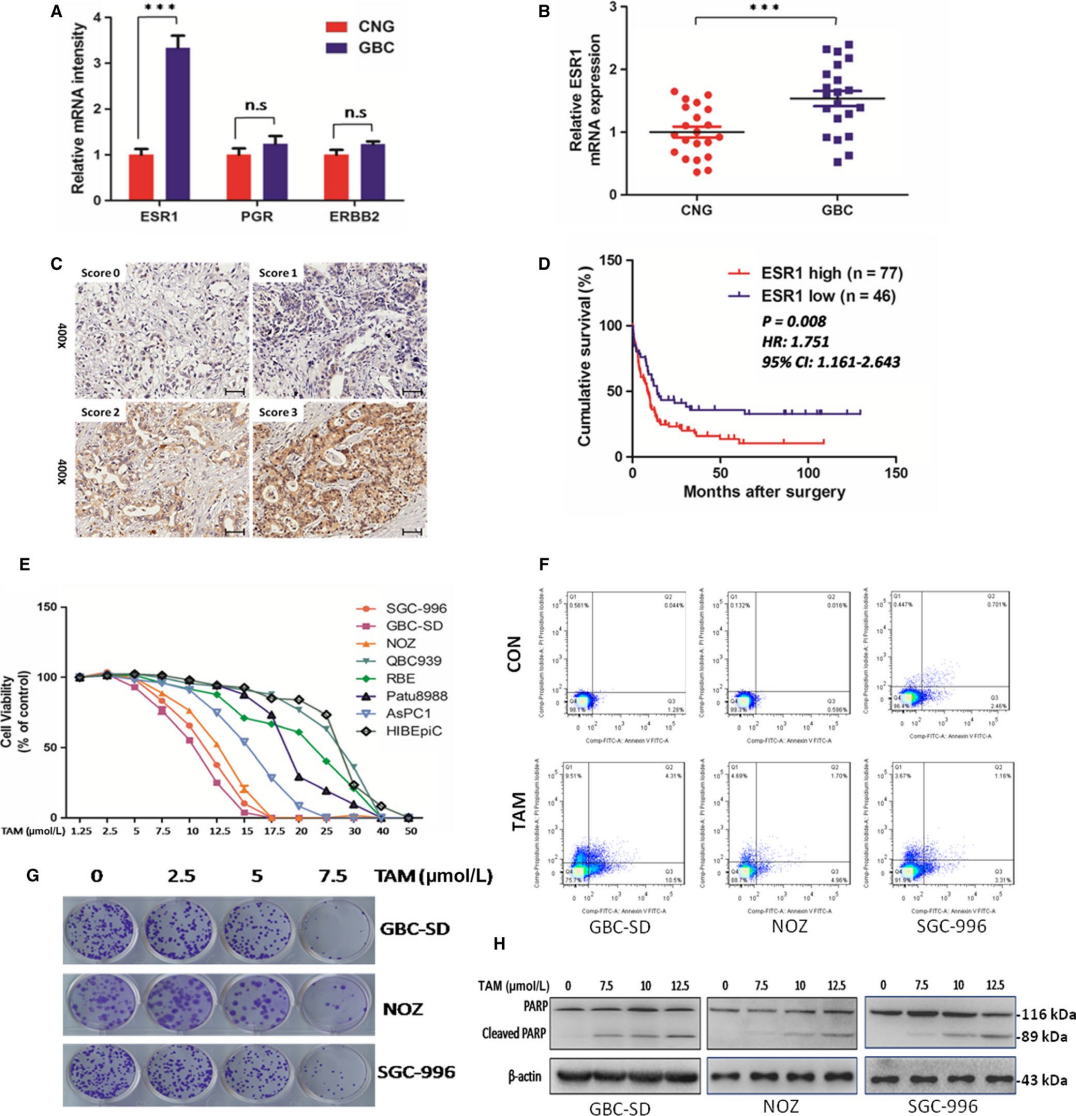
Altered ERa signalling is closely associated with gallbladder carcinogenesis, poor prognosis and a therapeutic target. A, Relative mRNA intensity of ERα, PGR and ERBB2 in mRNA expression array of six pairs of CNG and GBC tissues. n = 6 bar, SEM; Student's *t* test. B, Validation of ERα mRNA differential expression in an independent cohort consisting of 21 pairs of CNG and GBC tissues, n = 21; bar, SEM, Student's *t* test. C, Representative IHC staining images of different scores, which were calculated by intensity and percentage of stained cells as described in the methods. Scale bars: 50 μm. D, Kaplan‐Meier analysis of GBC patient survival. *P*‐value was calculated by log‐rank test. E, Cell viability in different cell lines treated with TAM for 48 h at indicated concentrations. GBC‐SD, SGC‐996 and NOZ are gallbladder cancer cell lines, QBC939 and RBE are cholangiocarcinoma cell lines, Patu8988 and AsPC1 are pancreatic carcinoma cell lines. HIBEpiC is human intrahepatic biliary epithelial cell line. F, Apoptosis rate analysis with Annexin V/PI flow cytometry in GBC‐SD,NOZ and SGC‐996 cells treated with TAM at 7.5 μmol/L for 48 h or without treatment. G, Representative images of colony in GBC cells. Cells were treated with TAM at 0, 2.5, 5 and 7.5 μmol/L for 24 h and incubated in the plate for 14 d. H, Protein levels of PARP and cleaved PARP. Cells were exposed to TAM at 0, 7.5, 10 and 12.5 μmol/L for 48 h before harvested for IB. TAM, tamoxifen, CNG, non‐cancerous gallbladder; GBC, gallbladder cancer. All n = 3; bar, SEM ***P < .001, β‐actin was the loading control in Western blot assay

In our RNA‐Seq study, six GBC patients were recruited and the expression level of ERɑ (ESR1), but not PR or HER2, was significantly increased (Figure 1A) in tumour tissue, which was further verified using QPCR in 21 paired cases (Figure 1B), suggesting ER signalling might be frequently altered in GBC. By evaluating ERɑ expression in 123 GBC cases using IHC (ERɑ low, score 0‐1 and ERɑ high, score 2‐3), we were able to found that increased level of ERɑ was correlated with worse clinical outcome, tumour size and TMN stages (Figure 1C,D and Table 1). Moreover, tumour size, histological grade, TNM stage, lymph node metastasis and distant metastasis were also risk factors for overall survival (OS). Besides, Cox model for multivariate analysis showed that only ERɑ was an independent prognostic factor for OS in GBC patients (Table 2). These results indicated patients conducting ERɑ high expression tended to have a poor prognosis.

**Table 1:** Correlation of ER expression with the clinicopathological characteristics of GBC

	ER high		ER low		*P*‐value
	N = 77	%	N = 46	%	
Sexual					
Male	20	26.0	14	30.4	.592
Female	57	74.0	32	69.6	
Age (y)					
<65	32	41.6	18	39.1	.791
≥65	45	58.4	28	60.9	
Tumour size (cm)					
<4	27	35.1	28	60.9	.005*
≥4	50	64.9	18	39.1	
Histological grade					
I‐II	42	54.5	26	56.5	.831
III‐IV	35	45.5	20	43.5	
TNM stage					
I‐II	25	32.5	24	52.2	.031*
III‐IV	52	67.5	22	47.8	
Lymph node metastasis					
No	53	68.8	32	69.6	.932
Yes	24	31.2	14	30.4	
Distant metastasis					
No	46	59.7	34	73.9	.111
Yes	31	40.3	12	26.1	

Note

*χ* ^2^ test was performed, *P* < .05 was considered statistically significant.

* *P* < .05.

**Table 2:** Univariate and multivariate analysis of the correlation of prognosis with ER and clinicopathologic data in GBC

	Univariable analysis		Multivariable analysis	
	HR (95% CI)	*P*‐value	HR (95% CI)	*P*‐value
Sex (male vs female)	0.748 (0.481‐1.164)	.199		
Age (<65 vs ≥65)	0.873 (0.582‐1.310)	.511		
Tumour size (≥4 cm vs <4 cm)	1.778 (1.173‐2.695)	.007*	1.427 (0.904‐2.252)	.126
Histological grade (III‐IV vs I‐II)	1.503 (1.004‐2.250)	.048*	1.487 (0.990‐2.234)	.056
TNM stage (III‐IV vs I–II)	1.946 (1.258‐3.010)	.003*	1.717 (0.997‐2.958)	.051
Lymph node metastasis (presence vs absence)	1.600 (1.049‐2.441)	.029*	1.106 (0.657‐1.859)	.705
Distant metastasis (presence vs absence)	1.806 (1.196‐2.728)	.005*	1.083 (0.606‐1.936)	.788
ER expression (high vs low)	1.799 (1.160‐2.790)	.009*	1.685 (1.071‐2.651)	.024*

Note

*P* < .05 was considered statistically significant.

Abbreviations: CI, confidence interval; HR, hazard ratio.

* *P* < .05.

Based on these findings, we speculate whether targeting ERɑ signalling would provide therapeutic effect for GBC. TAM, a clinical drug for breast cancer, showed cytotoxic effect against numerous cancer types including GBC cells. Treatment of GBC cells with TAM led to significant growth inhibition, reduced colony formation and marked apoptosis as analysed by Annexin V‐FITC/PI staining (Figure 1E‐G). Furthermore, increased cleavage of PARP was found in GBC cells receiving TAM therapy (Figure 1H). Although there was no significant difference among the half‐maximal inhibitory concentration (IC50) of GBC‐SD, NOZ and SGC‐996 cells, it seems GBC cells were more sensitive than cholangiocarcinoma and pancreatic cancer cell lines, indicating a possible relation with oestrogen/ER signalling. Expression of ERɑ and ERβ in cell lines showed chemoresistance to TAM was mainly related to ERɑ levels (Figure S1). GBC cells with ERɑ knockdown also exhibited stronger resistance to TAM, which further illustrated ERɑ was necessary for effects of TAM (Figure S2). Taken together, these data suggested targeting ERɑ by TAM might be effective for GBC.

### 3.2 TAM promoted GBC cell apoptosis by inducing ROS production via Nrf2 and CYPs

**Figure 2:**
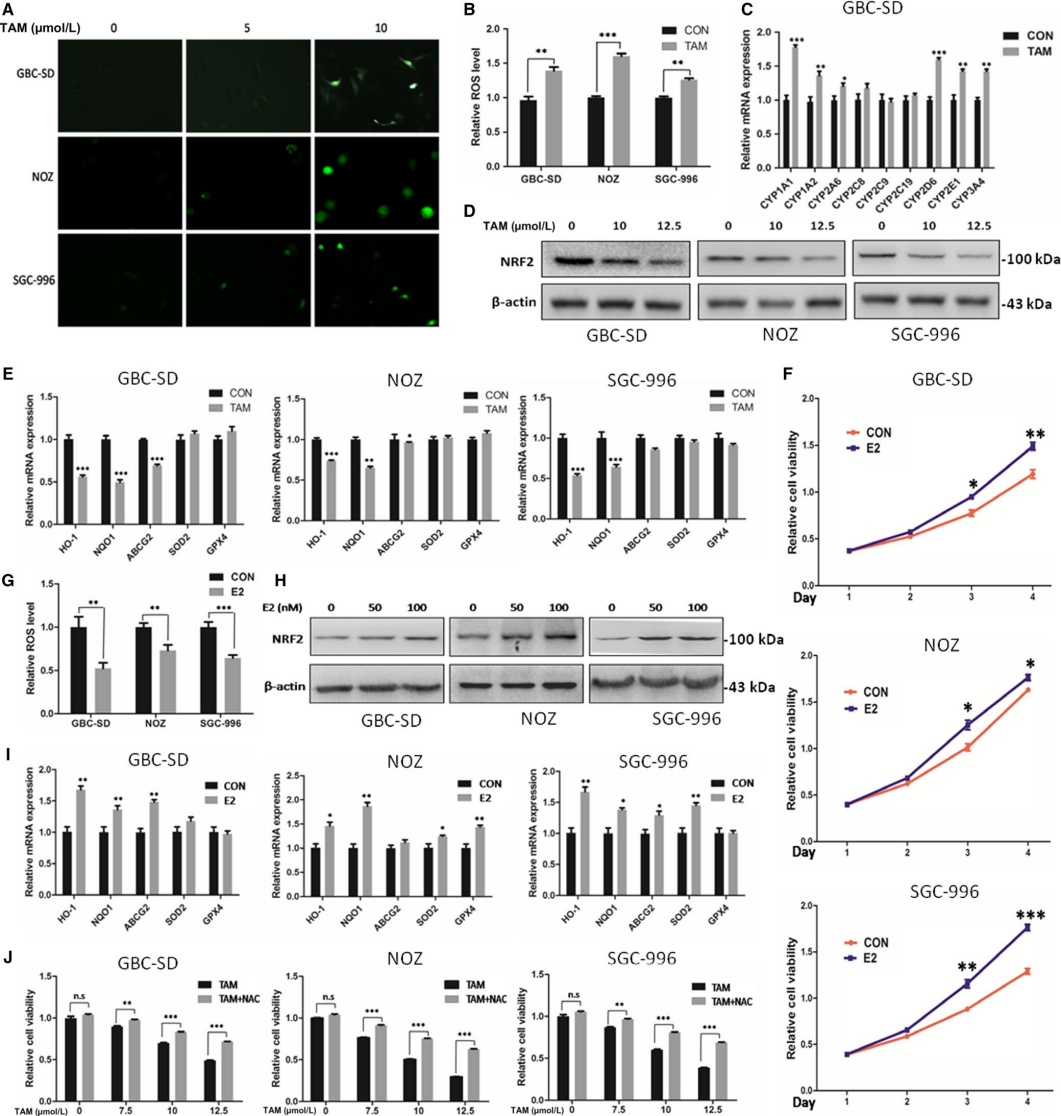
TAM promoted GBC cell apoptosis by inducing ROS production via Nrf2 and CYPs. A, Representative images of ROS levels in GBC‐SD, NOZ and SGC‐996 cells loaded with DCFDA. Cells were treated with TAM at 7.5 μmol/L for 48 h. B, Flow cytometry analysis of ROS levels in GBC‐SD, NOZ and SGC‐996 cells treated with TAM at 7.5 μmol/L for 48 h. C, The mRNA levels of CYP1A1, CYP1A2, CYP2A6, CYP2C8, CYP2C9, CYP2C19, CYP2C6, CYP2E1 and CYP3A4 in GBC‐SD were quantified by Q‐PCR. D, Protein level of NRF2 in GBC cells treated with TAM at 0, 10 and 12.5 μmol/L for 24 h. E, The mRNA levels of HO‐1, NQO1, ABCG2, SOD2 and GPX4 in GBC‐SD,NOZ and SGC‐996 cells treated with TAM at 12.5 μmol/L for 24 h. F, Cell viability in GBC‐SD,NOZ and SGC‐996 cells treated with E2 at 200 nmol/L for 72 h. G, Flow cytometry analysis of ROS levels in GBC‐SD,NOZ and SGC‐996 cells treated with E2 at 100 nmol/L for 24 h. H, Protein level of NRF2 in GBC cells treated with E2 at 0, 50 and 100 nmol/L for 24 h. I, The mRNA levels of HO‐1, NQO1, ABCG2, SOD2 and GPX4 in GBC‐SD,NOZ and SGC‐996 cells treated with E2 at 100 nmol/L for 24 h. J, Cell viability in GBC‐SD, NOZ and SGC‐996 cells treated with TAM/NAC co‐treatment for 48 h. TAM, tamoxifen; E2, 17β‐estradiol; and NAC, N‐acetyl‐L‐cysteine. GAPDH was used to normalize the Q‐PCR data, and β‐actin was used as control in Western blot assay. All n = 3; bar, SEM, NS, not significant, *P < .05; **P < .01; ***P < .001; Student's *t* test

Previously, we and other group have demonstrated the intracellular ROS level served as an important determinant for GBC chemoresistance.^18^ Most chemotherapeutic drugs induce oxidative stress associated with ROS generation and apoptosis.^19^ We thus determine whether TAM induces ROS in GBC cells for its pro‐apoptotic property. TAM increased the intracellular ROS levels in a dose‐dependent manner (Figure 2A). This result was further verified by flow cytometry analysis of cellular DCFH‐DA fluorescence (Figure 2B). Cytochrome P450 proteins (CYPs) are known to be involved in ROS production and drug metabolism. We next verify whether CYPs were implicated in TAM‐induced ROS production, and we found certain CYPs including CYP1A1, CYP2D6, CYP2E1, CYP1A2 and CYP2A6, were indeed increased when treated with TAM in GBC‐SD (Figure 2C and Figure S4), indicating TAM might promote ROS generation by selectively reprogramming CYPs expression profile. Additionally, Nrf2, a master regulator of redox homoeostasis, was previously reported to crosstalk with oestrogen signalling, we thus tested if TAM would modulate Nrf2 for impaired antioxidant response.^20^ Interestingly, GBC cells with TAM treatment showed reduced levels of Nrf2 and the mRNA transcripts of its target genes, NQO1 and HO‐1 (Figure 2D,E). Consistently, we found estradiol (E2), the most active oestrogen targeting ERα, was able to enhance GBC cell viability, inhibit ROS production, upregulate Nrf2 expression and activate most of its downstream targets (Figure 2F‐I). Upregulated expression of ERα and pS2 after E2 treatment indicated observed effects of E2 were through ERα activation (Figure S3B‐D). Finally, we determine whether ROS production was required for the pro‐apoptotic function of TAM in GBC cells. GBC cells were incubated with TAM alone or together with NAC, a potent scavenger of ROS. In contrast to TAM alone, the killing effect of TAM was remarkably attenuated upon NAC treatment, suggesting ROS was indispensable for the killing effect of TAM, at least partially (Figure 2J). Collectively, these data here have clearly indicated TAM enables GBC apoptosis by inducing ROS production partially through inactivation of Nrf2 signalling and increased expressions of CYPs.

### 3.3 TAM suppresses GBC viability via impaired glycolysis

**Figure 3:**
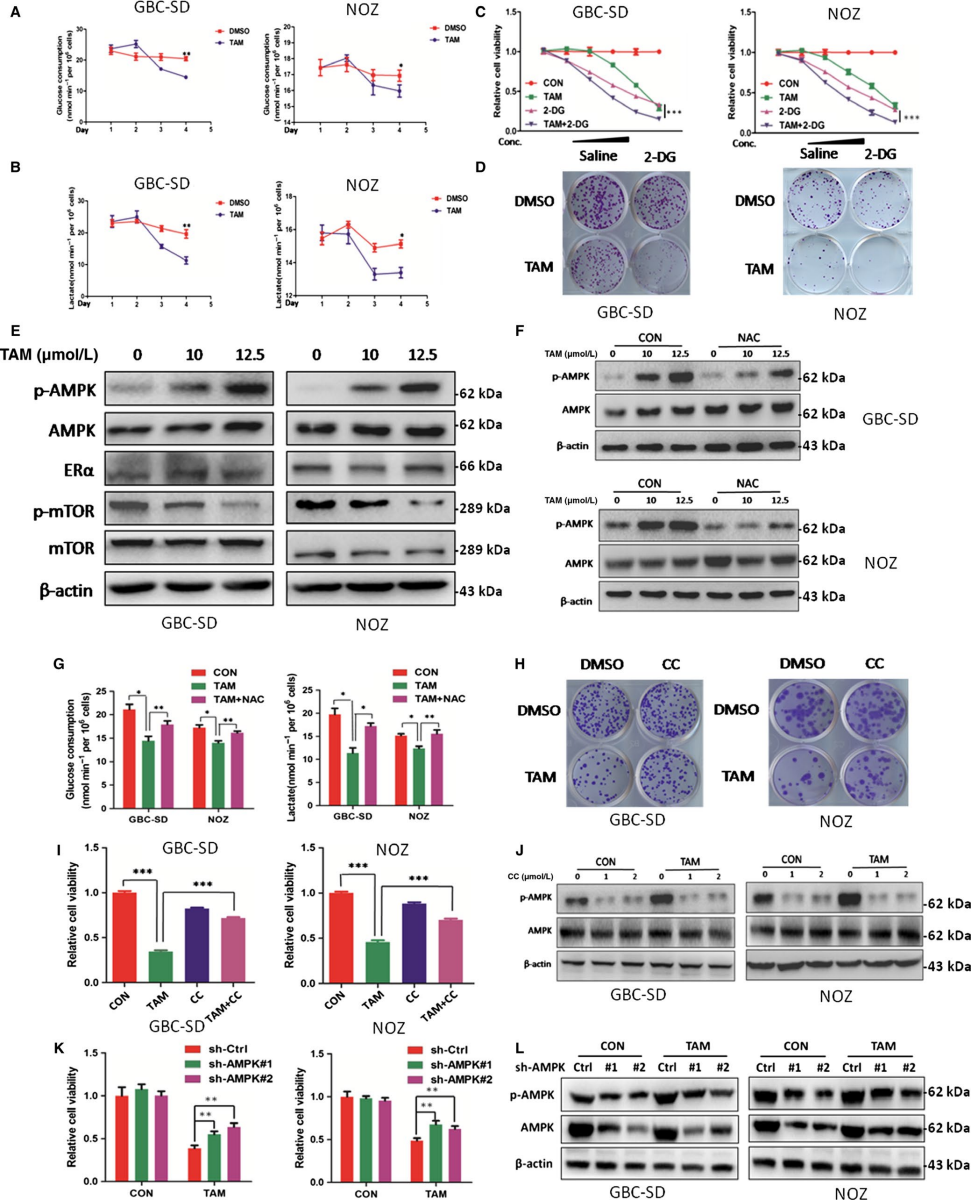
TAM suppressed glycolysis by activating AMPK signalling in a ROS‐dependent manner and induced GBC cell apoptosis. A, Glucose consumption of GBC cells treated with or without TAM at 7.5 μmol/L for 72 h. B, Lactate production of GBC cells treated with or without TAM at 7.5 μmol/L for 72 h. C, Cell viability in GBC‐SD and NOZ cells treated with TAM alone, 2‐DG alone and TAM/2‐DG combination for 48 h. TAM concentrations: 0, 5, 7.5, 10, 12.5, 15 μmol/L; 2‐DG concentrations: 0, 2.5, 5, 10, 20 and 40 mmol/L. D, Representative images of colony in GBC‐SD and NOZ cells. Cells were treated with TAM alone (5 μmol/L), 2‐DG alone (5 mmol/L) and TAM/2‐DG combination for 24 h and incubated in the plate for 14 d. E, Protein levels of p‐AMPK, total AMPK,ERα, p‐mTOR and mTOR in GBC cells treated with TAM at 0,10 and 12.5 μmol/L for 48 h. F, Protein level of p‐AMPK and total AMPK in GBC cells treated with TAM at 0, 10 and 12.5 μmol/L with or without NAC at 2 mmol/L for 48 h. G, Glucose consumption and lactate production of GBC cells treated with TAM alone or TAM/NAC co‐treatment for 48 h. H, Representative images of colony in GBC cells. Cells were treated with TAM alone (7.5 μmol/L), CC alone (1 μmol/L) and TAM/CC combination for 24 h and incubated in the plate for 14 d. I, Cell viability in GBC‐SD and NOZ cells treated with TAM alone (12.5 μmol/L), CC alone (2 μmol/L) and TAM/CC combination for 48 h. J, Protein levels of p‐AMPK and total AMPK in GBC cells treated with TAM at 0 and 12.5 μmol/L with or without CC for 48 h. K, Cell viability in GBC cells with or without AMPK knockdown treated with TAM (0 and 12.5 μmol/L) for 48 h. L, Protein levels of p‐AMPK and total AMPK in GBC cells treated with TAM at 0 and 12.5 μmol/L with or without AMPK knockdown for 48 h. TAM, tamoxifen; 2‐DG, 2‐deoxy‐D‐glucose; NAC, N‐acetyl‐L‐cysteine; and CC, compound C. β‐actin was used as control in Western blot assay. All n = 3; bar, SEM, *P < .05; **P < .01; ***P < .001; Student's *t* test

Given that aerobic glycolysis is critical for rapid tumour growth and provide tumour‐specific survival advantages,^21^ we next investigated whether this suppressive effect of TAM was mediated through modulation of glycolysis. GBC cells treated with non‐lethal dose of TAM for 48 hours or longer led to decreased glucose consumption and lactate production, indicating impaired glycolysis (Figure 3A,B). Interestingly, reduced glycolysis was found during the time periods over 48 hours while slight increased glycolysis was observed within 24 hours. It is quite reasonable because plenty of studies indicated low concentration TAM treatment show slight oestrogen‐like activity in short time.^22^, ^23^

Since TAM could inhibit cell growth by inhibiting glycolysis, we examine whether 2‐DG, an glycolysis inhibitor, would facilitate the function of TAM in GBC cells. Expectedly, 2‐DG with TAM significantly reduced cell growth and colony formation ability and showed a synergetic role of 2‐DG and TAM in promoting GBC cell death (Figure 3C,D). These findings suggested TAM induces GBC apoptosis via glycolysis remodelling.

### 3.4 TAM suppresses glycolysis by activating AMPK signalling in a ROS‐dependent manner

Except for its function in cellular damage, ROS serves as a signalling molecule and enacts a wide range of biological functions including glycolysis through modifications of lipids, nucleic acids and proteins.^24^ As a major metabolic regulator, AMPK is activated in numerous types of cancer cells under excessive stress and contributes to cell apoptosis and death.^25^ Multiple studies indicated AMPK contains redox‐sensitive cysteines that are rapidly oxidized during oxidative stress and could inhibit glycolysis.^26^, ^27^, ^28^ We hypothesized that TAM might activate AMPK signalling in GBC through a redox‐dependent mechanism.

Gallbladder cancer cells treated with TAM indeed induced AMPK activation, which was accompanied by decreased phosphorylation of mTOR (p‐mTOR) (Figure 3E), a critical regulator of cancer cell glycolysis,^29^, ^30^ indicating impaired glycolysis. To determine whether the effect of glycolysis inhibition by TAM was dependent on the upstream activation of ROS, we measured the level of p‐AMPK in GBC cells treated with TAM alone or along with NAC. As shown in Figure 3F, addition of NAC significantly attenuated the phosphorylation of AMPK induced by TAM. Importantly, NAC also recovered the glucose uptake and the production of lactate down‐regulated by TAM treatment, which were consistent with the effect of NAC on TAM‐induced apoptosis (Figure 3G). Together, these data provide evidence that TAM promoted GBC apoptosis through impaired glycolysis via ROS production. Experiments of SGC‐996 were shown in Figure S5.

### 3.5 TAM inhibited GBC cells by activating AMPK signalling

Since we have demonstrated TAM suppresses glycolysis via activation of AMPK, compound C (AMPK inhibitor) and AMPK knockdown were used to further evaluate whether the AMPK signalling pathway is required for TAM‐induced suppression of GBC cells. As shown, AMPK inhibitor compound C (CC) reversed the pro‐apoptotic effect of TAM (Figure 3H,I). In lines with the effect, CC dramatically abrogated AMPK phosphorylation (Figure 3J).GBC cells with AMPK knockdown also exhibited stronger resistance to TAM (Figure 3K,L). Taken together, these data indicated that TAM inhibited GBC cell growth largely by activating AMPK signalling. Experiments of SGC‐996 were shown in Figure S6.

### 3.6 TAM enhances CDDP‐induced inhibition of cell viability in GBC cells

**Figure 4:**
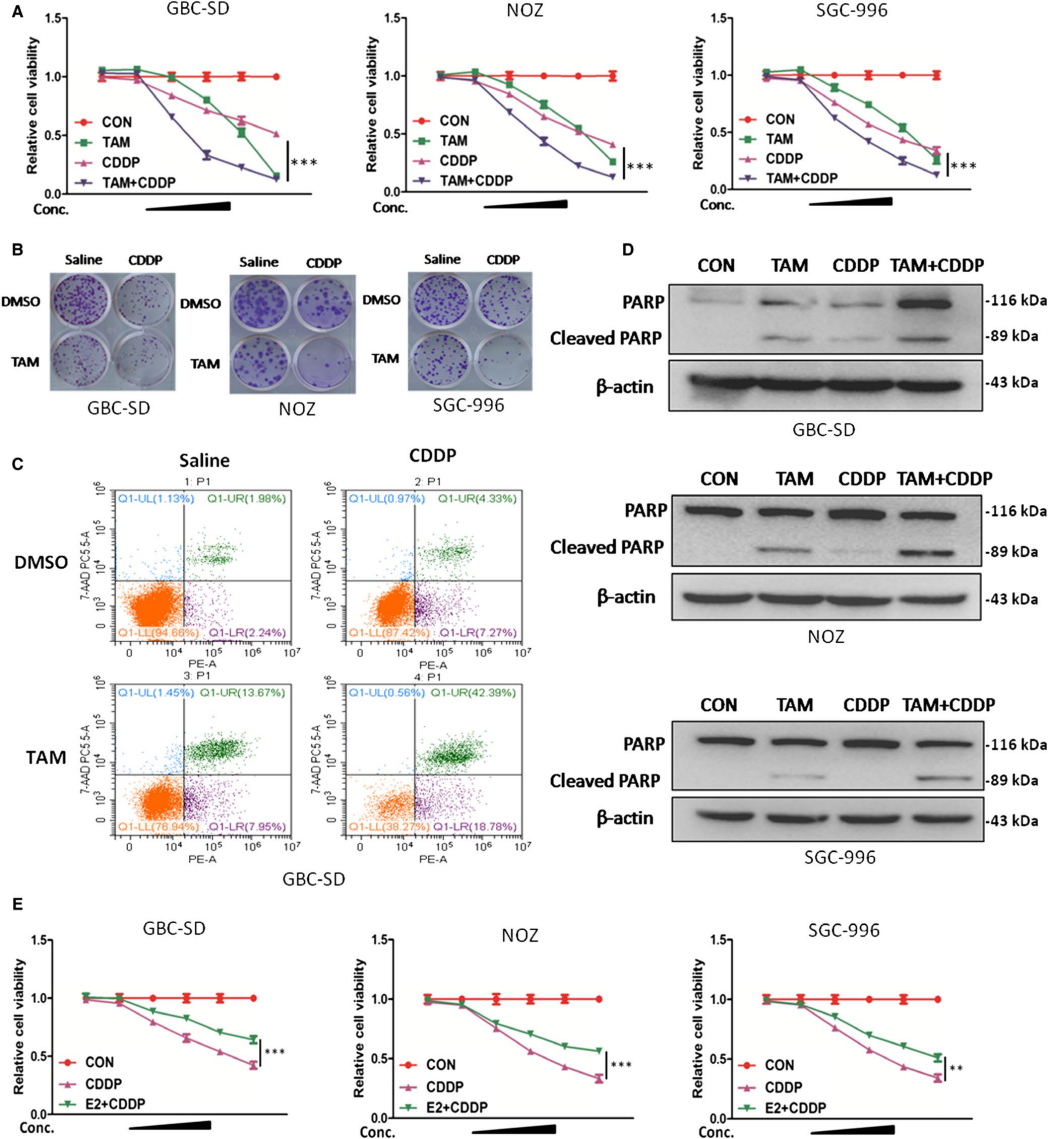
TAM enhanced CDDP‐induced inhibition of cell viability in GBC cells. A, Cell viability in GBC‐SD,NOZ and SGC‐996 cells treated with TAM alone, CDDP alone, and TAM/CDDP combination for 48 h. TAM concentrations:0, 5, 7.5, 10, 12.5 and 15 μmol/L; CDDP concentrations: 0, 1, 2, 4, 8 and 12 μm. B, Representative images of colony in GBC cells. Cells were treated with TAM alone (5 μmol/L), CDDP alone (2 μmol/L) and TAM/CDDP combination for 24 h and incubated in the plate for 14 d. C, Apoptosis rate analysis using PE/7‐AAD flow cytometry in GBC‐SD cells treated with TAM alone (12.5 μmol/L), CDDP alone (4 μmol/L), and TAM/CDDP combination for 48 h. D, Protein levels of PARP and cleaved PARP in GBC cells treated with TAM alone (12.5 μmol/L), CDDP alone (4 μmol/L) and TAM/CDDP combination for 48 h. E, Cell viability in GBC‐SD,NOZ and SGC‐996 cells treated with E2 alone, CDDP alone, and E2/CDDP combination for 48 h. E2 concentrations: 0, 50, 100, 200, 400 and 800 nmol/L; CDDP concentrations: 0, 1, 2, 4, 8 and 12 μmol/L. TAM, tamoxifen; CDDP,cisplatin; E2, 17β‐estradiol,β‐actin was used as control in Western blot assay. All n = 3; bar, SEM

Chemoresistance is a major obstacle to the chemotherapeutic efficacy of GBC. Discovery of novel agent that boosts the functions of first‐line drugs would provide improved survival benefits for patients with GBC. It is therefore interesting to examine whether TAM has synergetic effect in enhancing CDDP therapeutic efficacy. GBC cell viability was then evaluated upon treatment with TAM and CDDP alone or in combination. We found the cell viability induced by TAM or CDDP alone was comparable while a combined use of TAM and CDDP in GBC cells resulted in more significant cell death as revealed by CCK8 and colony formation ability (Figure 4A,B). The synergetic effect seems more evident in GBC‐SD and NOZ cells. These results suggested that co‐treatment of TAM and CDDP possessed a synergistic effect on GBC cell growth. Cell apoptosis analysis using FACS further supported that CDDP‐induced apoptosis was markedly enhanced by TAM in GBC cells (Figure 4C). Consistently, cleaved PARP was also found to be markedly increased in response to TAM/CDDP co‐treatment in contrast to CDDP or TAM alone (Figure 4D). No change in LC3 expression was found after TAM or/and CDDP treatment (Figure S6D). Collectively, these data indicate co‐treatment with TAM significantly enhanced apoptosis induced by CDDP.

As part of evidence, we also verified whether E2 would have the opposite effect on CDDP. A remarkable apoptotic effect was observed in cells treated with CDDP alone compared with CDDP/E2 combination (Figure 4E). Taken together, these findings suggested targeting ERɑ indeed sensitized CDDP therapeutic effect.

### 3.7 TAM effectively sensitizes the tumour xenografts to CDDP in vivo

**Figure 5:**
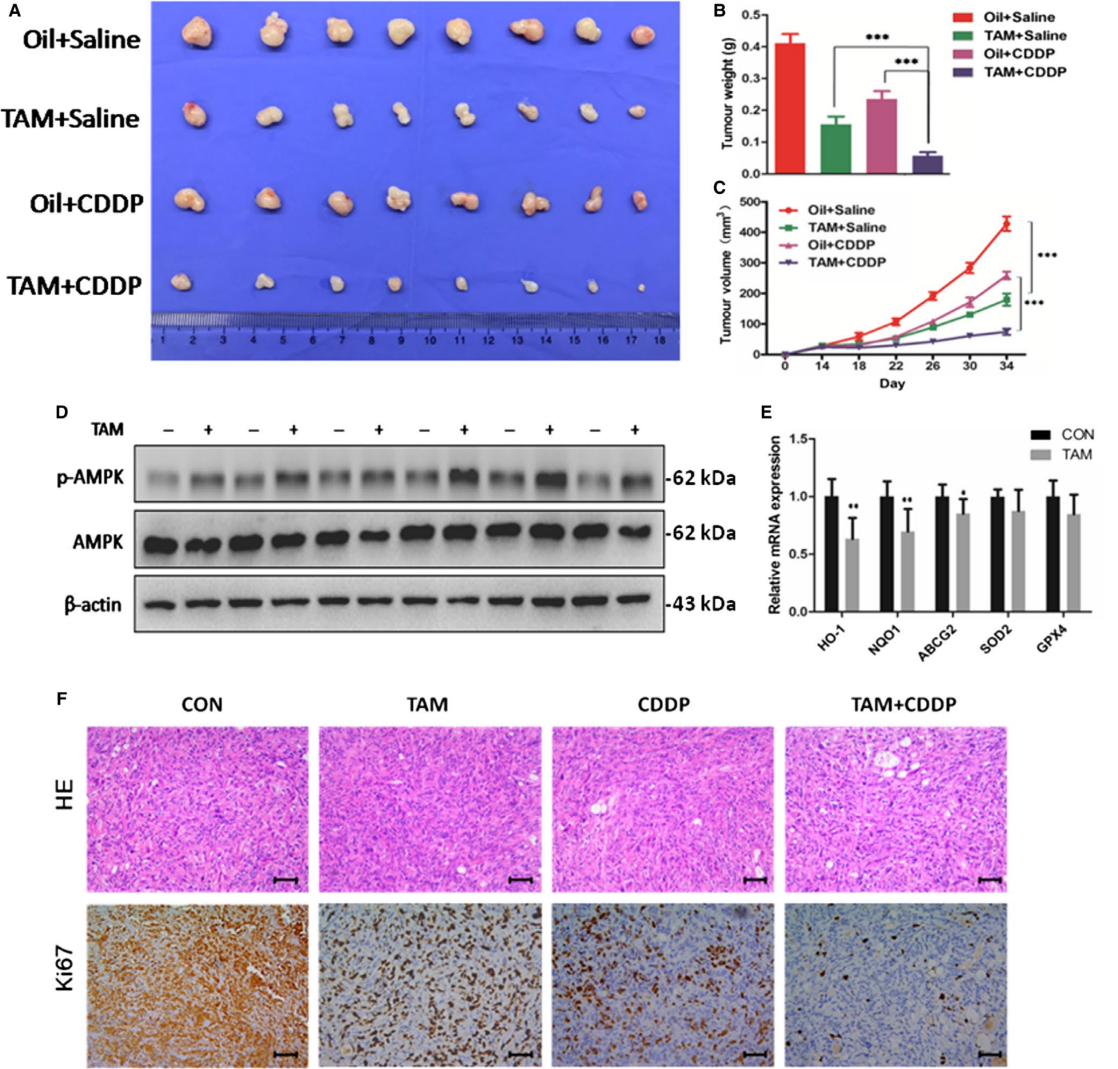
TAM markedly sensitized the tumour xenografts to CDDP cytotoxicity in vivo. The tumour ‐bearing mice were injected intraperitoneally with dissolvents, TAM alone, CDDP alone and TAM/CDDP co‐administration. A, Photograph of transplanted tumours after the mice were exposed to treatments (n = 8/group). Oil and peanut oil. B, Tumour weight of four groups after the mice was exposed to treatments. C, Tumour growth curves of GBC‐SD cells after treatment in vivo. D, Western blot to analyse p‐AMPK and AMPK protein expression in transplanted tumours. E, Q‐PCR to analyse HO‐1, NQO1, ABCG2, SOD2 and GPX4 mRNA levels in transplanted tumours. F, H&E staining and Ki67 immunostaining in transplanted tumour tissues. Scale bars: 50 μm. TAM, tamoxifen; CDDP, cisplatin; Q‐PCR data were normalized by GAPDH, and β‐actin was used as control in Western blot assay. All bar, SEM, *P < .05; **P < .01; ***P < .001; Student's *t* test

Our experiments in vitro showed that drug resistance of GBC cells could be markedly overcome by TAM in combination with CDDP. To further study the synergistic effect of TAM and CDDP in vivo, GBC‐SD cells were injected into male nude mice subcutaneously. Two weeks later, mice were randomly separated into four equal groups. The tumour‐bearing mice were intraperitoneally injected with vehicle control, TAM, CDDP or TAM‐CDDP combination for approximately 20 days. Treatment of mice with TAM or CDDP alone both inhibited tumour growth, but TAM‐CDDP combination treatment resulted in a significant reduction of the average tumour weight (Figure 5A,B). Tumour growth curve data further indicated the potent anticancer effect in the TAM‐CDDP combined group (Figure 5C).

To examine the effect of TAM treatment on AMPK and NRF2 downstream signalling in vivo, Western blot and Q‐PCR were performed using the tumour xenografts. As shown in Figure 5D, p‐AMPK was significantly increased in tumours obtained from TAM‐treated mice as compared with those from vehicle control mice. At the same time, down‐regulated transcriptional levels of HO‐1, NQO1 and ABCG2 were observed after TAM treatment (Figure 5E). Lower Ki67 was induced by CDDP or TAM treatment, and in particular, more significantly by TAM/CDDP combined treatment (Figure 5F). These results were in agreement with results in vitro.

Pathological changes in major organs were measured to evaluate systemic toxic effects of the treatments for these mice. No obvious differences were observed between the treated groups (Figure S7). Collectively, these results demonstrated that tumour growth was effectively inhibited by TAM‐CDDP co‐treatment without obvious toxic effects in vivo.

### 3.8 Ovariectomy promoted sensitivity of tumour xenografts to CDDP cytotoxicity

**Figure 6:**
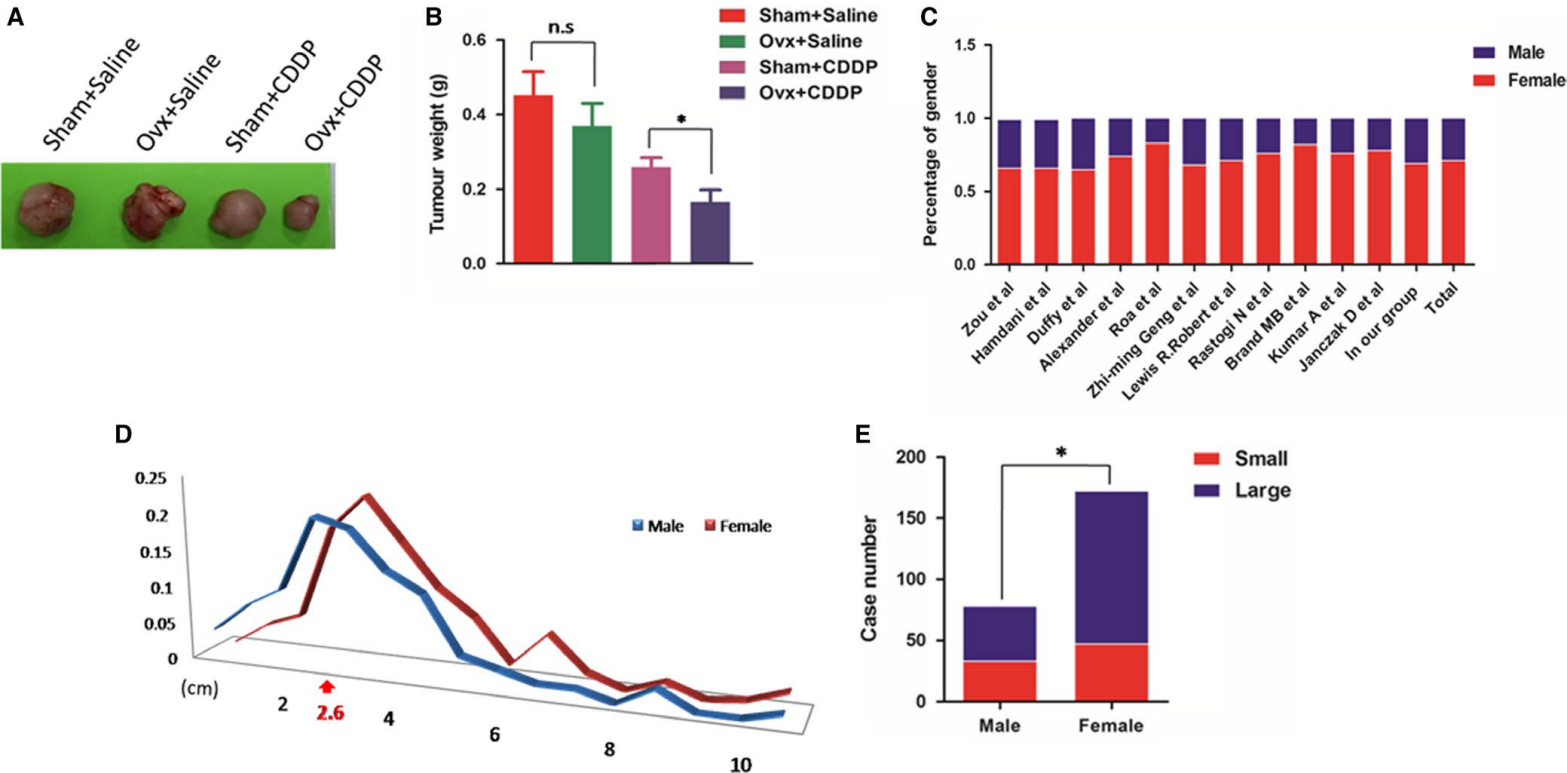
Ovariectomy promoted sensitivity of tumour xenografts to CDDP cytotoxicity. A, B, Representative images and tumour weight of the four groups (n = 5/group), Sham, sham operation; Ovx, ovariectomy. All bar, SEM, NS, not significant, *P < .05; Student's *t* test. C, Gender distribution of gallbladder cancer in different studies including our group. D, E, Distribution of tumour size (diameter) by gender. Curve intersection: 2.6 cm. *P < .05, *χ* ^2^ tests

To identify ER signalling effect on the growth and drug resistance of gallbladder cancer cells, we designed ovariectomy group as showed and observed that both growth and drug resistance were affected by ovariectomy in vivo. The effect on the drug resistance of gallbladder cancer is very significant (Figure 6A,B). The average weight of tumours in the ovariectomized group obviously decreased. But because of the large difference intra the group, the statistical difference is not significant (Figure 6B).

### 3.9 Clinical analysis exhibited higher morbidity and larger tumour size in female patients of GBC

Given that gallbladder cancer is highly associated with gender, we collected relevant studies and patients in our hospital, and found that gallbladder cancer incidence is indeed higher in women, accounting to approximately 60%‐80% (Figure 6C),^31^ suggesting a strong association with ER signalling. In our following analysis of clinic data, distribution of tumour size by gender further hinted promoting role of ER signalling in gallbladder cancer (Figure 6D,E).

## 4 DISCUSSION

**Figure 7:**
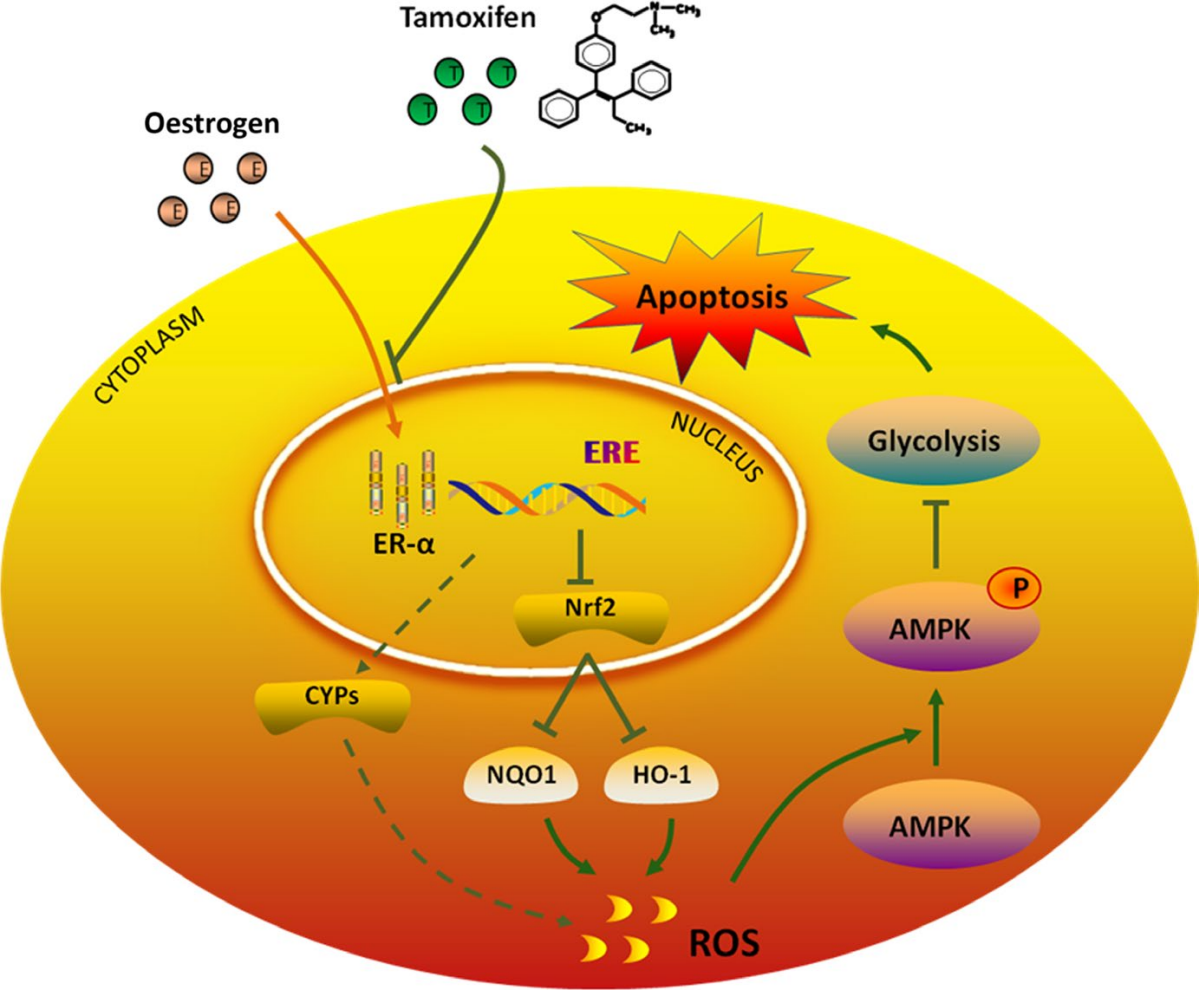
A hypothetical schematic representation of the pro‐apoptotic effects of TAM on GBC cells. TAM can bind to ERɑ competitively and trigger ROS accumulation by down‐regulating Nrf2 and increasing CYPs expression, which subsequently stimulates GBC apoptosis via aerobic glycolysis inhibition by activating AMPK signalling

Rapid progression and chemotherapy resistance directly lead to poor prognosis of GBC.^32^, ^33^ Increasing evidence has shown that gallbladder cancer is closely related to oestrogen. High levels of endogenous oestrogen and acquisition of exogenous oestrogen are all shown to exert a critical role on the incidence and prognosis of GBC.^10^, ^34^, ^35^ While ER signalling seems to be vital for gallbladder cancer, attention has not been paid to the current clinical treatment.^36^ TAM is a classical oestrogen receptor antagonist widely used in the treatment of breast cancer,^11^, ^37^ while roles of TAM in GBC are still lacking (Figure 7).

In our study, we found the expression of ERα is upregulated in GBC tissues and associated with worse prognosis, implying altered ERα signalling might provide therapeutic values for GBC. Indeed, TAM was effective against several GBC cell lines. Notably, the IC50 of GBC cell seemed lower than cholangiocarcinoma and pancreatic cancer cell lines, indicating a possible relation with oestrogen/ER signalling.

Chemoresistance was reported to be associated with elevated level of ROS in tumours including GBC. We thus determine whether TAM induces ROS in GBC cells. TAM increased the intracellular ROS levels dose‐dependently, suggesting TAM might induce GBC apoptosis via ROS generation. Given that Nrf2 serves a master regulator of oxidative stress, we thus tested if TAM was involved in regulation of Nrf2 for ROS production. Interestingly, GBC cells with TAM treatment showed reduced levels of Nrf2 and the mRNA transcripts of its target genes, HO‐1 and NQO1, whereas E2 displayed the opposite effect. Additionally, some CYPs were found to be increased in response to TAM in GBC‐SD, possibly contributing to ROS accumulation. Furthermore, we determine NAC, a potent scavenger of ROS was observed to remarkably attenuated TAM activity, suggesting ROS was required for the cytotoxic effect of TAM, at least partially. Collectively, these data here demonstrated TAM promoted GBC apoptosis by induction of ROS.

Multiple researches showed aerobic glycolysis is active in various cancers and critical for tumour survival under energy stress and hypoxia,^21^, ^38^, ^39^ which activates ROS production. More importantly, ROS is involved not only for its direct cellular damage, it serves important messenger in a broad range of biological processes through modifications of lipids, nucleic acids and proteins,^40^ while AMPK, a master energy sensor, was reported to be activated by ROS during hypoxia. These evidences led us to determine whether this suppressive effect of TAM was mediated through modulation of glycolysis via AMPK signalling triggered by ROS. TAM was indeed found to activate AMPK signalling, inhibit mTOR and glycolysis, thereby limiting GBC growth and inducing apoptosis. In combined treatment of TAM and glycolysis inhibitor 2‐DG, we also found glycolysis inhibition would facilitate the function of TAM in GBC cells. These findings indicated potential role of metabolic regulation in GBC treatment.^41^

Recent study revealed that AMPK could be activated by hypoxia in absence of AMP via ROS‐dependent CRAC channel activation, resulting in increasing cytosolic calcium which activate the AMPK upstream kinase CaMKKβ.^26^, ^42^ Besides, ROS can activate AMPK directly via oxidative modification or glutathionylation of the AMPKα subunit.^43^ It has also been suggested that ROS can activate LKB1/AMPK pathway via involving a cytoplasmic form of the PI‐3 kinase‐like kinase, ataxia‐telangiectasia mutated (ATM).^44^ Whether AMPK activation in present study is regulated by TAM through this mechanism remained to be further investigated. Notably, TAM was observed to show an oestrogenic effect at low concentration within short‐time treatment, which is consistent with previous studies in other groups. It is interpretable that TAM might have some metabolites with oestrogen‐like action at the oestrogen receptor.^23^ Future studies should expand research efforts on comprehensive understandings of TAM functions in cancers.

Finally, we demonstrated combined therapy of TAM and CDDP was significantly effective for GBC in vivo. Interestingly, we found the ablation of oestrogens by ovariectomy in nude mice strikingly increased CDDP sensitivity, suggesting ERɑ is required for overcoming chemoresistance. In our following analysis of clinic data, distribution of gender and tumour size further hinted important regulatory role of oestrogen receptor signalling in gallbladder cancer.

It is notable that the unique pharmacokinetic characteristics make tamoxifen more advantageous in gallbladder cancer treatment.^45^ Tamoxifen metabolites 4‐hydroxytamoxifen and N‐desmethyl‐4‐hydroxytamoxifen, which possess a much stronger ER inhibitory activity,^46^ could be secreted in bile and participate in enterohepatic circulation.^47^, ^48^, ^49^ The bile infiltrating environment of GBC provides another platform for tamoxifen metabolites to reach tumour. Enterohepatic circulation also prolongs the half‐life of tamoxifen and increases drug efficacy.^47^

Some studies suggested ERBB2 would impair effect of TAM in breast cancer.^50^, ^51^ Recently, a study indicated ERBB2 mutations are the most extensively mutated pathway in GBC and were associated with poor prognosis in individuals.^52^ New ERBB2 inhibitors development in GBC is carrying out and will help TAM to achieve a better efficacy.^53^ Various endocrine therapies would be indispensable for GBC treatment in the future.

Taken together, our findings in vitro and in vivo indicated that targeting ERɑ signalling by TAM provides improved therapeutic values for GBC, and sheds novel light on the clinical use of endocrine therapy for patients with GBC.

## CONFLICT OF INTEREST

The authors confirm that there are no conflicts of interest.

## AUTHOR CONTRIBUTIONS

Jian Wang designed the research study and wrote the paper; Shuai Huang, Hui Wang, Xince Huang performed the research; Wei Chen and Ming Zhan contributed essential reagents; Shuai Huang, Ruirong Lin, Sunwang Xu and Hui Shen analysed the data.

## Data Availability

The data that support the findings of this study are available from the corresponding author upon reasonable request.
