# Supplementary figures and images for: Tamoxifen inhibits cell proliferation by impaired glucose metabolism in gallbladder cancer
Source: J Cell Mol Med. 2019 Nov 28;24(2):1599–613. doi: 10.1111/jcmm.14851 (PMC6991689; doi:10.1111/jcmm.14851)

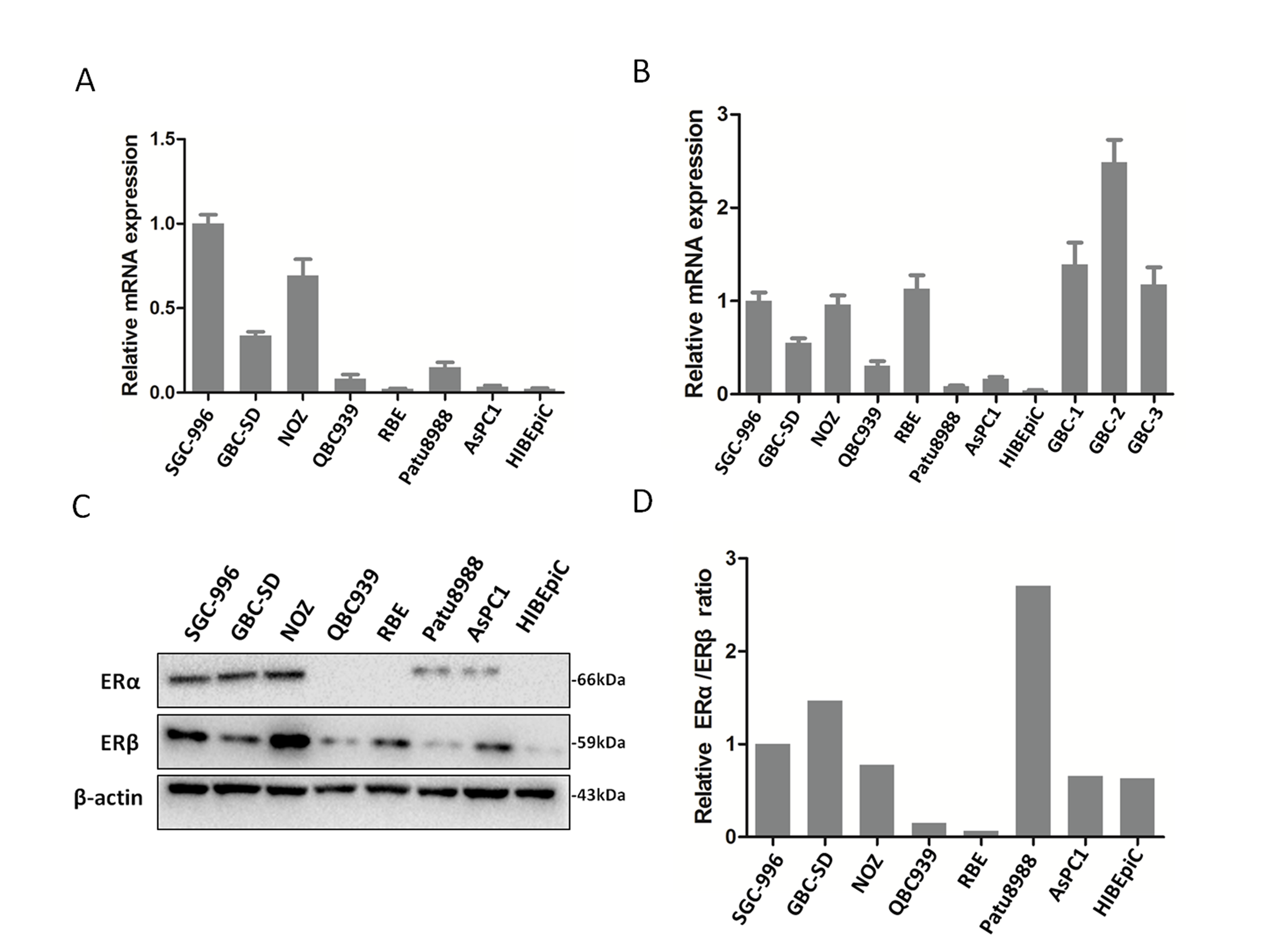

Supplement: Supplementary file 1 [file JCMM-24-1599-s001.tif]

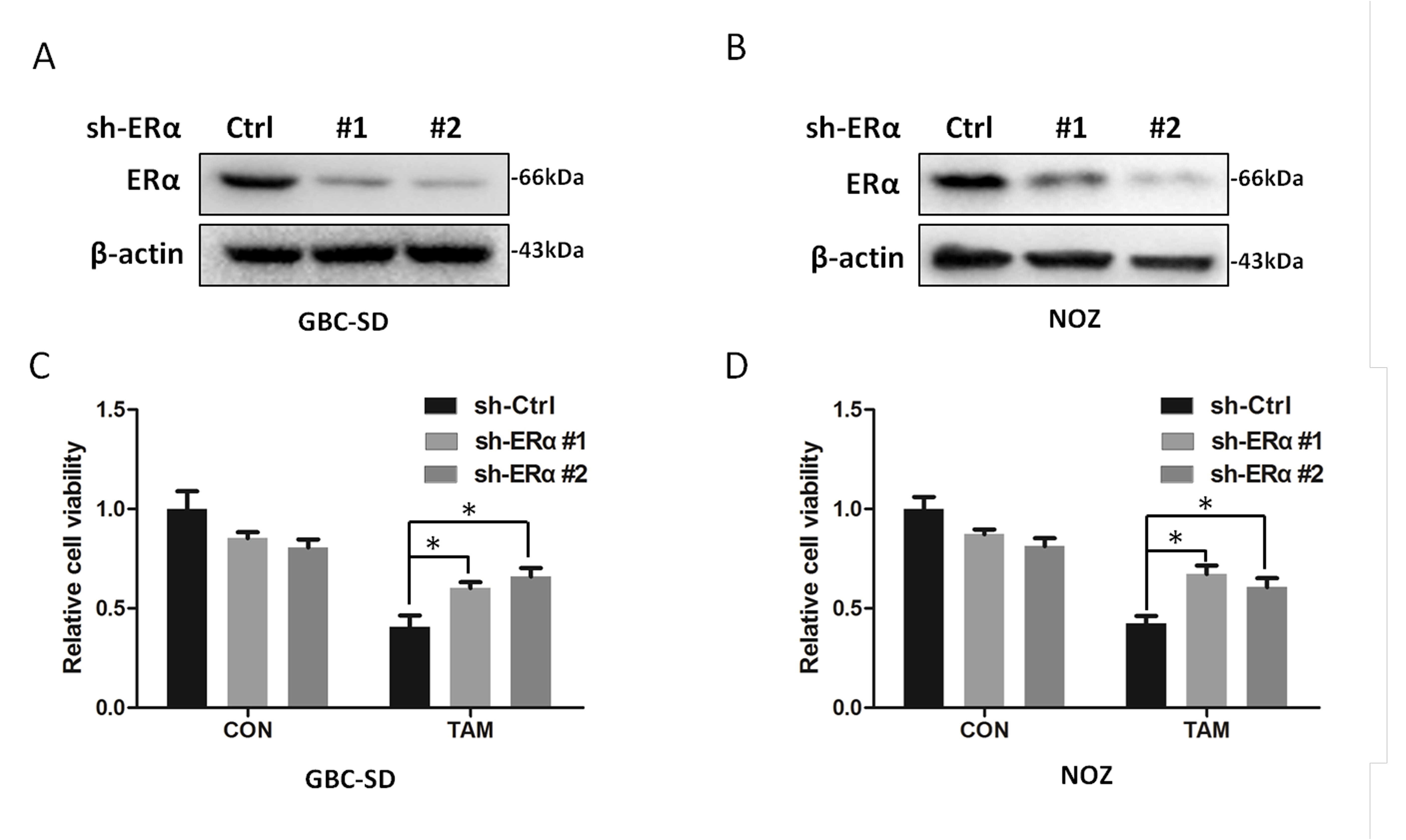

Supplement: Supplementary file 2 [file JCMM-24-1599-s002.tif]

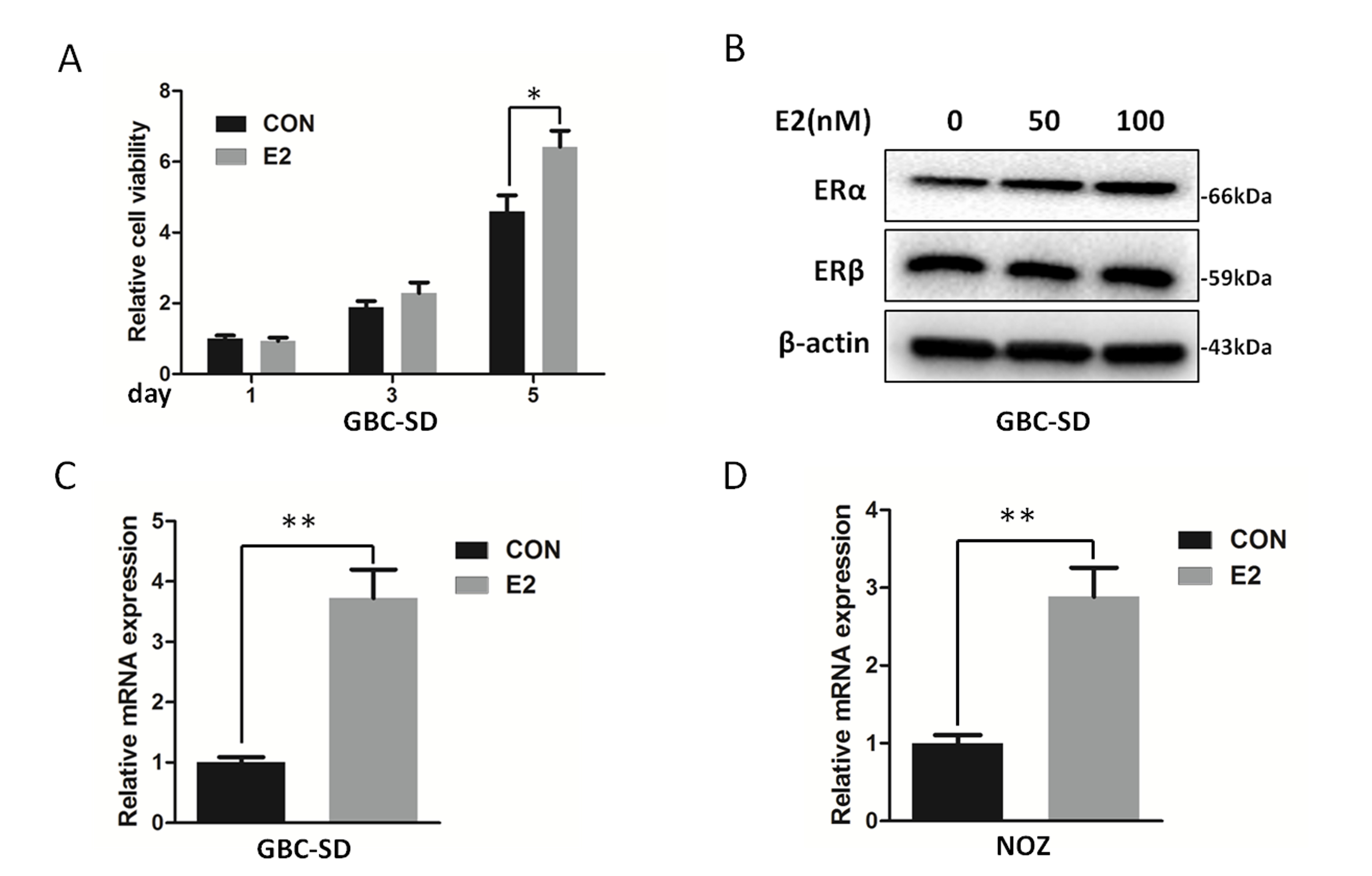

Supplement: Supplementary file 3 [file JCMM-24-1599-s003.tif]

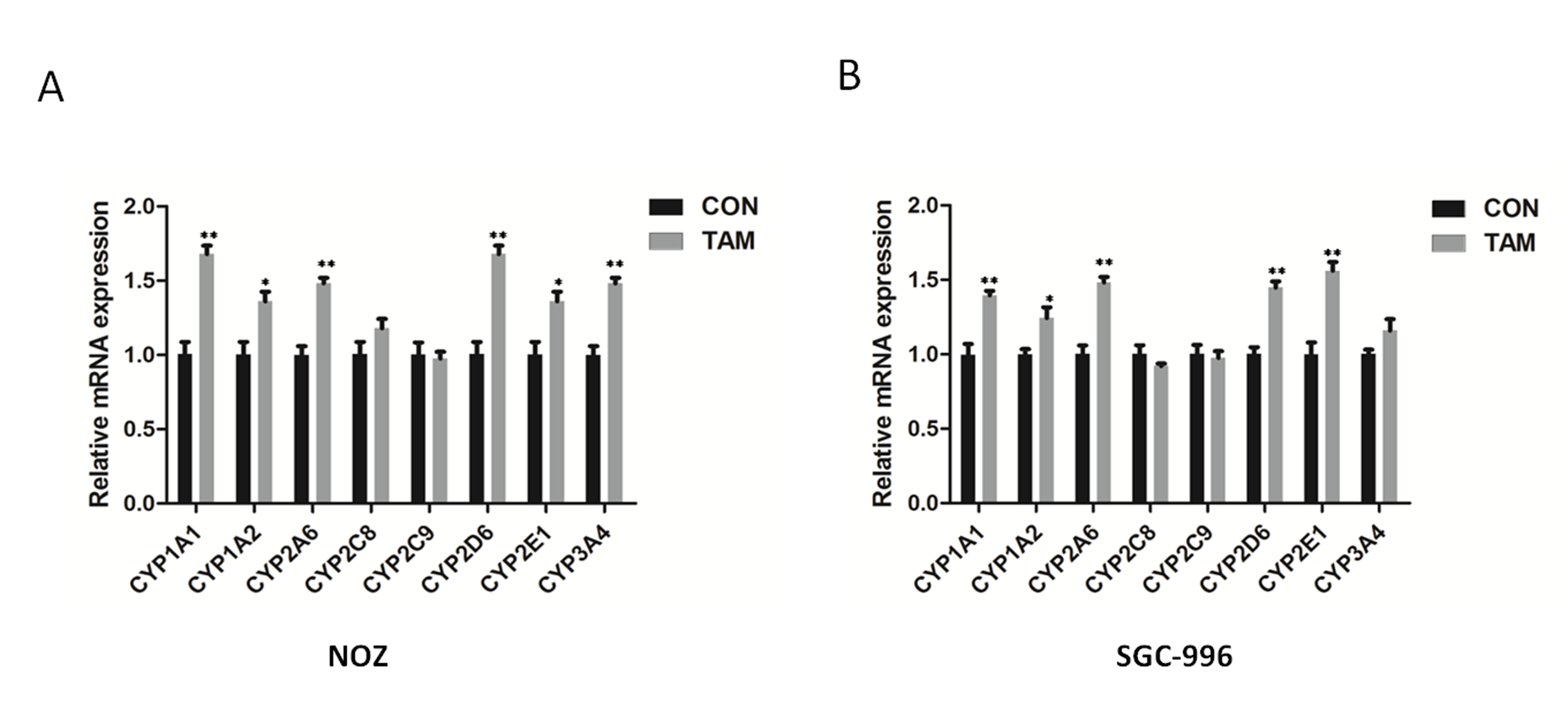

Supplement: Supplementary file 4 [file JCMM-24-1599-s004.tif]

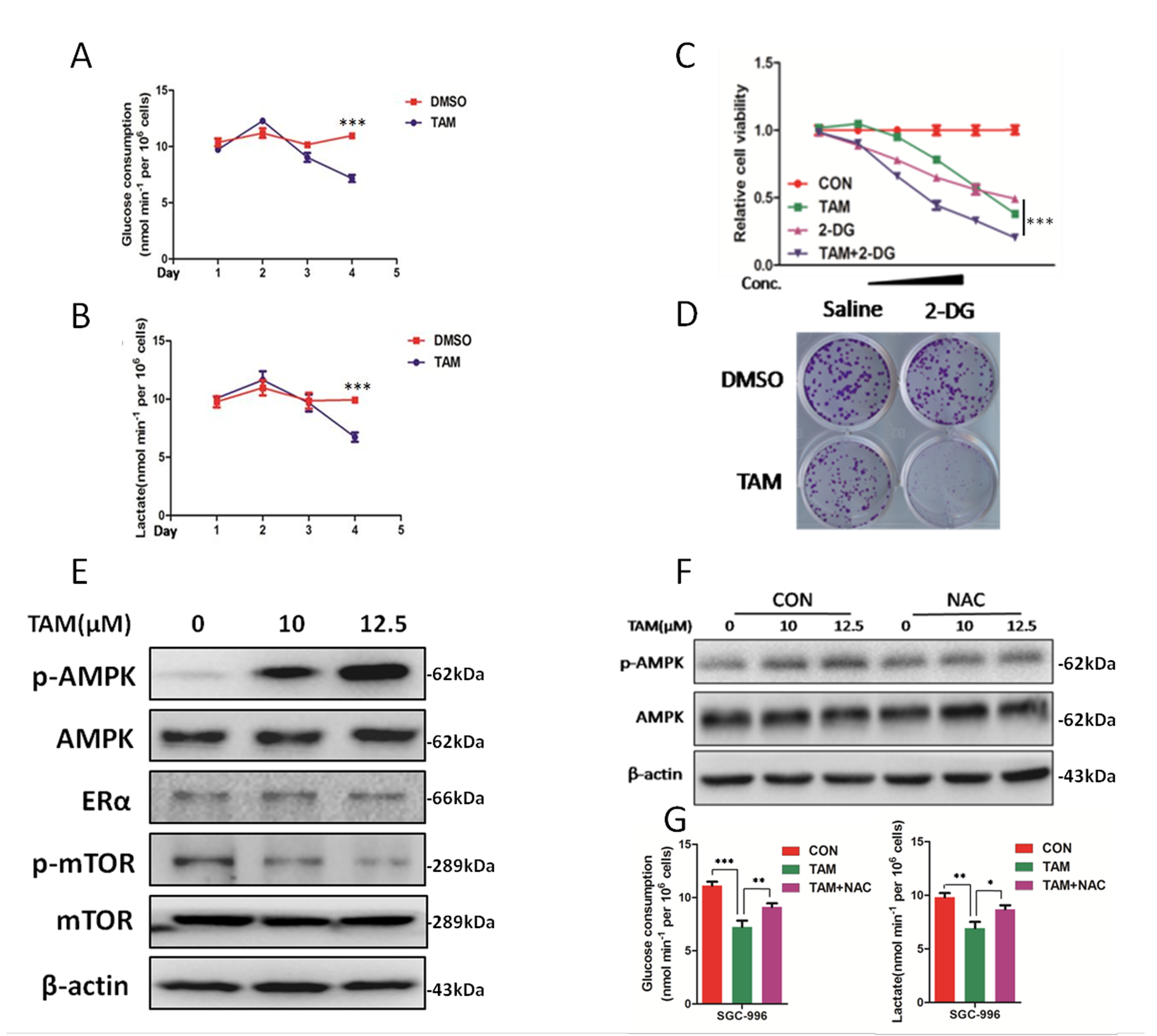

Supplement: Supplementary file 5 [file JCMM-24-1599-s005.tif]

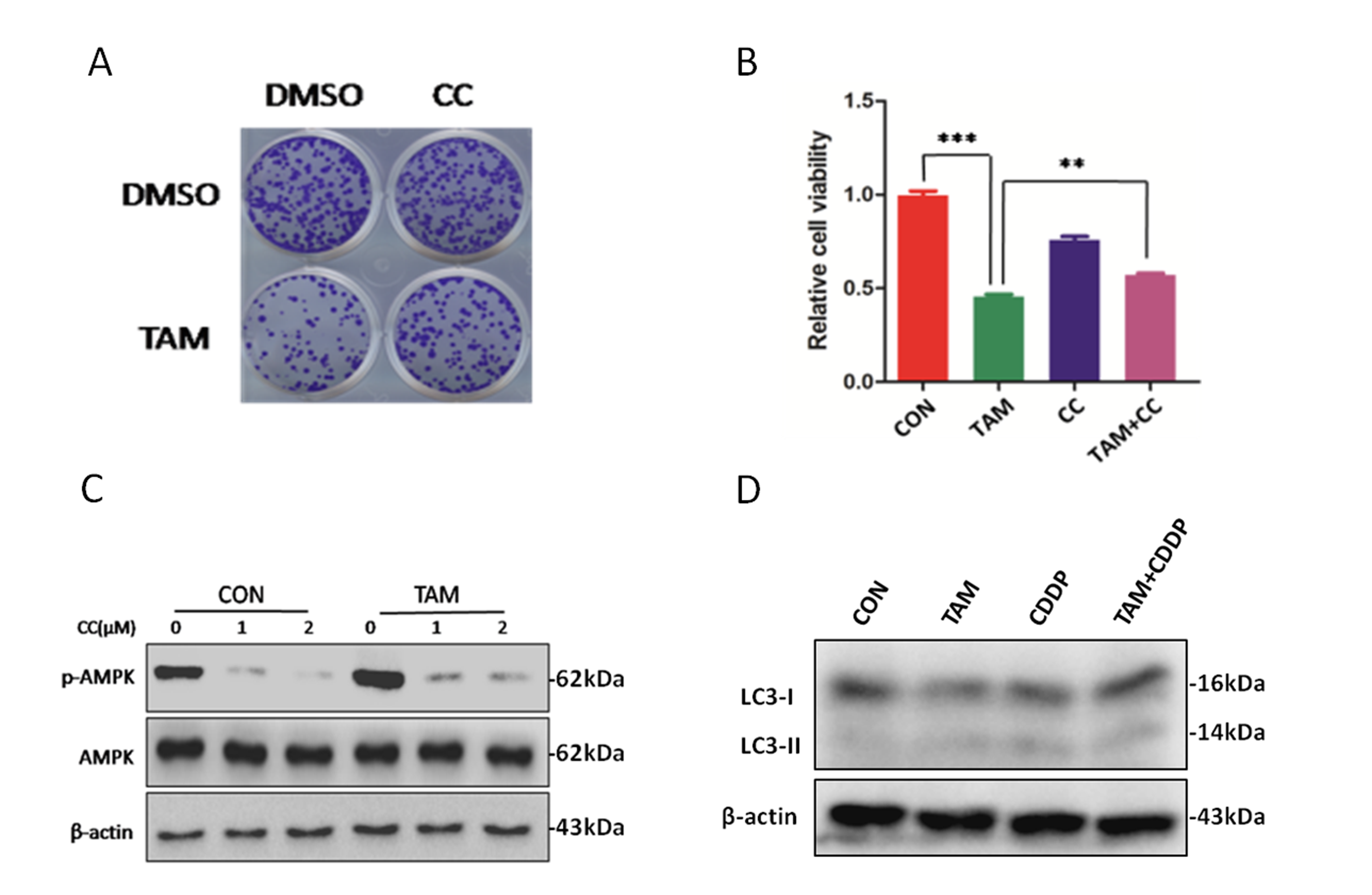

Supplement: Supplementary file 6 [file JCMM-24-1599-s006.tif]

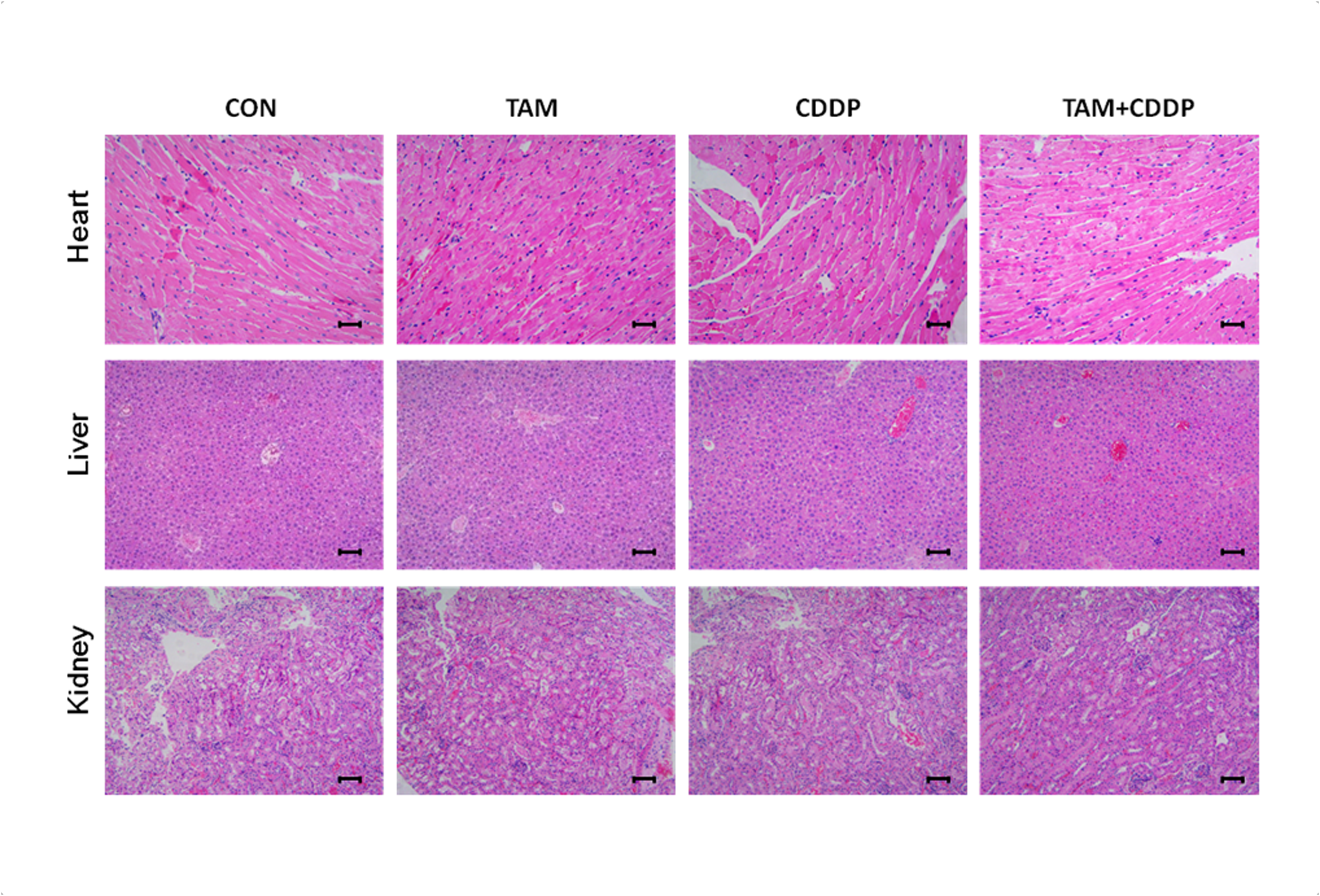

Supplement: Supplementary file 7 [file JCMM-24-1599-s007.tif]

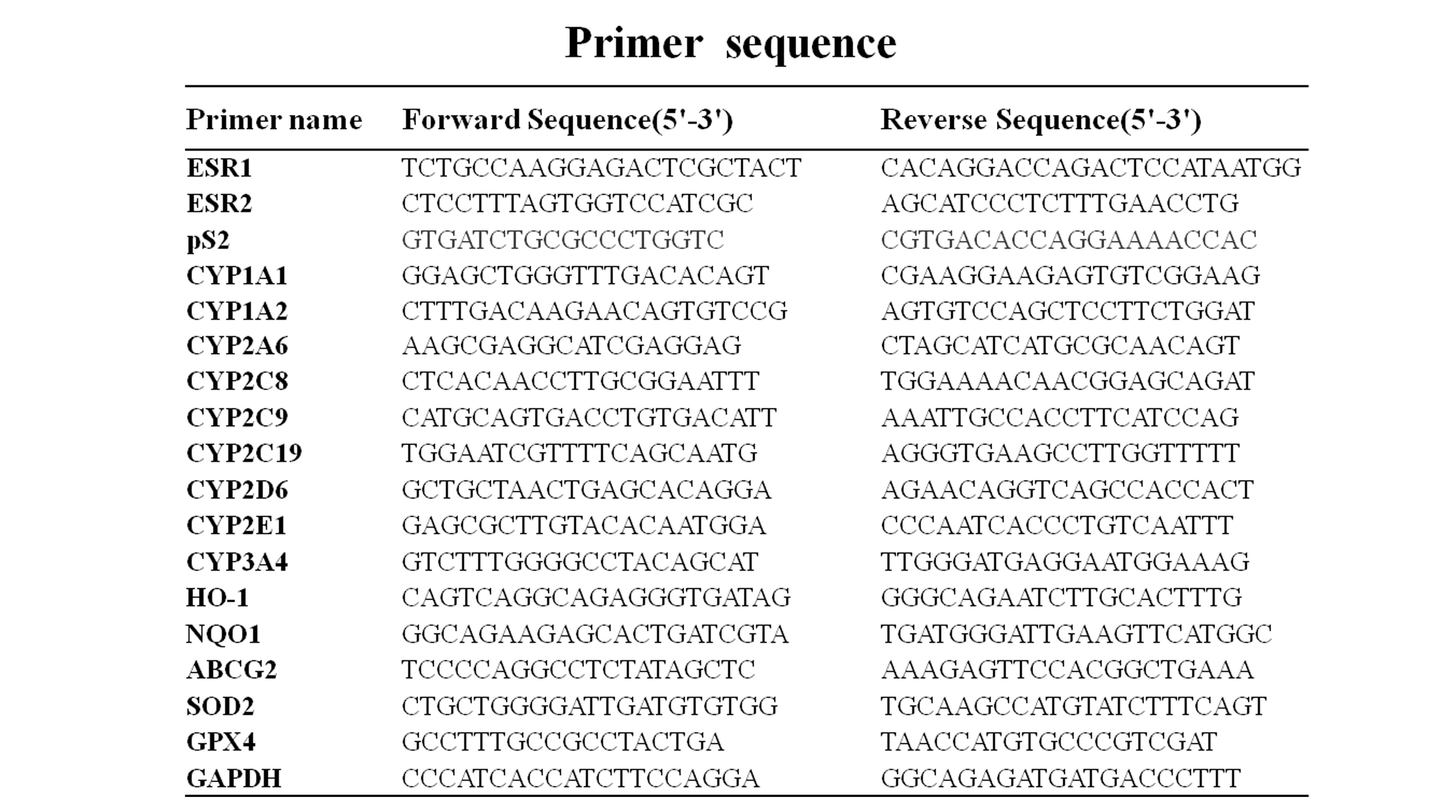

Supplement: Supplementary file 8 [file JCMM-24-1599-s008.tif]
