# Supplementary material for: Tamoxifen inhibits cell proliferation by impaired glucose metabolism in gallbladder cancer
Source: J Cell Mol Med. 2019 Nov 28;24(2):1599–613. doi: 10.1111/jcmm.14851 (PMC6991689; doi:10.1111/jcmm.14851)
Supplement: Supplementary file 9 [file JCMM-24-1599-s009.pdf]

No.	Gender	Age	Hormonal therapy	Menopause	Contraceptive pills	Treatment choice
1	Female	53	/	√	/	Surgery
2	Male	77	/	/	/	/
3	Male	51	/	/	/	Surgery
4	Female	75	/	√	/	/
5	Male	68	/	/	/	Surgery
6	Female	51	/		/	Surgery
7	Female	68	/	√	/	/
8	Male	70	/	/	/	Surgery
9	Female	66	/	√	/	Surgery
10	Female	55	/		/	/
11	Female	42	/		√	Surgery
12	Female	51	/		/	Surgery
13	Female	69	/	√	/	Surgery
14	Female	64	/	√	/	Surgery
15	Male	61	/	/	/	Surgery
16	Female	79	/	√	/	Surgery
17	Female	54	/	√	/	/
18	Female	51	Estradiol Valerate	√	/	Surgery
19	Female	91	/	√	/	Surgery
20	Female	57	/	√	/	Surgery
21	Female	70	/	√	/	Surgery
22	Male	74	/	/	/	/
23	Male	83	/	/	/	Surgery
24	Female	73	/	√	/	Surgery
25	Male	79	/	/	/	/
26	Female	50	/		/	Surgery
27	Male	71	/	/	/	Surgery
28	Female	58	/	√	/	Surgery
29	Male	59	/	/	/	Surgery
30	Male	69	/	/	/	Surgery
31	Male	67	/	/	/	/
32	Female	84	/	√	/	Surgery
33	Male	75	/	/	/	Surgery
34	Female	52	/		/	/
35	Female	55	/		/	Surgery
36	Female	68	/	√	/	Surgery
37	Female	42	/		/	/
38	Male	79	/	/	/	Surgery
39	Female	64	/	√	/	Surgery
40	Male	80	/	/	/	Surgery
41	Male	59	/	/	/	/
42	Male	62	/	/	/	Chemotherapy
43	Male	48	/	/	/	/
44	Female	49	/		/	Surgery
45	Female	58	/	√	/	Surgery
46	Female	48	/		/	Surgery
47	Female	74	/	√	/	Surgery
48	Male	50	/	/	/	Surgery
49	Female	44	/		√	Surgery

50	Female	64	/	√	/	/
51	Female	51	/		/	/
52	Female	77	/	√	/	Surgery
53	Female	76	/	√	/	Surgery
54	Male	65	DES for prostatic cancer	/	/	Surgery
55	Female	71	/	√	/	Surgery
56	Female	76	/	√	/	Surgery
57	Female	62	/	√	/	/
58	Female	75	/	√	/	Surgery
59	Male	75	/	/	/	Surgery
60	Female	83	/	√	/	Surgery
61	Female	65	/	√	/	Surgery
62	Female	75	/	√	/	Surgery
63	Female	72	/	√	/	Surgery
64	Male	55	/	/	/	Surgery
65	Male	64	/	/	/	Chemotherapy
66	Male	62	/	/	/	Surgery
67	Male	52	/	/	/	/
68	Male	60	/	/	/	Surgery
69	Male	55	/	/	/	/
70	Male	63	/	/	/	Surgery
71	Male	58	/	/	/	/
72	Female	78	/	√	/	Surgery
73	Female	57	/		/	Surgery
74	Female	61	/	√	/	Surgery
75	Male	61	/	/	/	Surgery
76	Male	61	/	/	/	Chemo/Radiotherapy
77	Male	64	/	/	/	Surgery
78	Male	38	DES after circumcision	/	/	/
79	Male	58	/	/	/	Surgery
80	Male	63	/	/	/	/
81	Male	69	/	/	/	Chemotherapy
82	Male	75	/	/	/	/
83	Female	44	/		/	Surgery
84	Female	78	/	√	/	Surgery
85	Male	85	/	/	/	/
86	Male	72	/	/	/	Radiotherapy
87	Male	69	/	/	/	/
88	Female	80	/	√	/	Surgery
89	Female	72	/	√	/	Surgery
90	Male	62	/	/	/	Surgery
91	Female	72	/	√	/	Surgery
92	Male	82	/	/	/	/
93	Male	73	/	/	/	Surgery
94	Male	71	/	/	/	Surgery
95	Male	69	/	/	/	/
96	Male	75	/	/	/	Surgery
97	Male	65	/	/	/	/
98	Male	74	/	/	/	Surgery
99	Male	78	/	/	/	/

100	Male	79	/	/	/	Surgery
101	Male	81	/	/	/	Radiotherapy
102	Male	68	/	/	/	/
103	Female	49	/		/	Surgery
104	Female	64	/	√	/	Surgery
105	Male	62	/	/	/	Surgery
106	Female	65	/	√	/	Surgery
107	Male	50	/	/	/	Surgery
108	Male	72	/	/	/	/
109	Male	77	/	/	/	Surgery
110	Male	66	/	/	/	/
111	Male	69	/	/	/	Surgery
112	Male	71	/	/	/	/
113	Male	79	/	/	/	Surgery
114	Male	67	/	/	/	/
115	Male	80	/	/	/	Surgery
116	Male	68	/	/	/	Surgery
117	Male	61	/	/	/	Surgery
118	Female	51	/		/	/
119	Female	51	Estradiol Valerate	√	/	Surgery
120	Female	50	/		/	/
121	Female	53	/		/	Surgery
122	Female	55	Estradiol Valerate	√	/	Chemotherapy
123	Female	54	/		/	Surgery
124	Female	49	/		/	/
125	Female	52	/		/	Surgery
126	Female	50	/		/	Surgery
127	Female	58	/	√	/	/
128	Female	48	/		/	Surgery
129	Female	44	/		/	/
130	Female	61	/	√	/	Surgery
131	Female	54	/		/	/
132	Female	57	/	√	/	Surgery
133	Female	49	/		/	/
134	Female	52	/		/	Chemotherapy
135	Female	41	/		/	/
136	Female	66	/	√	/	Surgery
137	Female	83	/	√	/	Surgery
138	Female	63	/	√	/	/
139	Male	72	/	/	/	Surgery
140	Female	41	/		/	Surgery
141	Female	63	/	√	/	Surgery
142	Male	47	DES after circumcision	/	/	Surgery
143	Female	83	/	√	/	Surgery
144	Female	83	/	√	/	Surgery
145	Female	49	/		/	/
146	Female	48	/		/	Surgery
147	Female	52	/		/	/
148	Female	61	/	√	/	Surgery
149	Female	60	/	√	/	/

150	Female	58	/	√	/	Surgery
151	Female	44	/		/	/
152	Female	49	/		/	Surgery
153	Female	46	/		/	Surgery
154	Female	38	/		/	/
155	Female	42	/		/	Surgery
156	Female	51	/		/	/
157	Female	62	/	√	/	Surgery
158	Female	79	/	√	/	Surgery
159	Female	71	/	√	/	Surgery
160	Female	65	/	√	/	Surgery
161	Male	63	/	/	/	Surgery
162	Female	80	/	√	/	Surgery
163	Female	49	/		/	Surgery
164	Female	75	/	√	/	Surgery
165	Female	70	/	√	/	Surgery
166	Male	33	DES after circumcision	/	/	Surgery
167	Female	71	/	√	/	Surgery
168	Female	56	/	√	/	Surgery
169	Female	72	/	√	/	Surgery
170	Female	46	/		/	/
171	Female	49	/		/	Surgery
172	Female	47	/		/	/
173	Female	54	/		/	Chemotherapy
174	Female	53	/		/	/
175	Female	68	/	√	/	Surgery
176	Female	57	/	√	/	Surgery
177	Female	75	/	√	/	Surgery
178	Female	49	/		/	Surgery
179	Female	58	/	√	/	/
180	Female	41	/		/	Surgery
181	Female	52	Estradiol Valerate	√	/	/
182	Female	44	/		/	Surgery
183	Female	54	/		/	Chemotherapy
184	Female	50	/		/	Surgery
185	Female	64	/	√	/	/
186	Female	43	/		/	Surgery
187	Female	55	/		/	Surgery
188	Female	67	/	√	/	/
189	Male	73	/	/	/	Surgery
190	Female	79	/	√	/	Surgery
191	Female	54	/	√	/	Surgery
192	Female	81	/	√	/	Surgery
193	Female	77	/	√	/	/
194	Male	65	/	/	/	Surgery
195	Female	52	/		/	Surgery
196	Male	59	/	/	/	Surgery
197	Female	79	/	√	/	Surgery
198	Female	77	/	√	/	Surgery
199	Female	74	/	√	/	/

200	Female	77	/	√	/	Surgery
201	Female	75	/	√	/	/
202	Female	84	/	√	/	Surgery
203	Female	69	/	√	/	Surgery
204	Female	60	/	√	/	Surgery
205	Female	87	/	√	/	Surgery
206	Male	59	/	/	/	Surgery
207	Female	55	/		/	Surgery
208	Female	90	/	√	/	/
209	Female	69	/	√	/	Surgery
210	Female	72	/	√	/	/
211	Female	83	/	√	/	Surgery
212	Female	67	/	√	/	/
213	Female	74	/	√	/	Surgery
214	Female	73	/	√	/	/
215	Female	55	/		/	Surgery
216	Female	65	/	√	/	Surgery
217	Female	69	/	√	/	Surgery
218	Male	73	/	/	/	Surgery
219	Female	63	/	√	/	Surgery
220	Female	69	/	√	/	/
221	Female	62	/	√	/	Surgery
222	Male	52	/	/	/	Surgery
223	Female	75	/	√	/	Surgery
224	Female	53	/		/	Surgery
225	Male	74	/	/	/	Surgery
226	Female	83	/	√	/	Surgery
227	Female	70	/	√	/	Surgery
228	Female	77	/	√	/	Surgery
229	Male	60	/	/	/	Surgery
230	Male	70	/	/	/	Surgery
231	Female	70	/	√	/	Surgery
232	Male	71	/	/	/	Surgery
233	Female	76	/	√	/	/
234	Female	69	/	√	/	Chemotherapy
235	Female	73	/	√	/	/
236	Female	76	/	√	/	Radiotherapy
237	Female	75	/	√	/	/
238	Female	77	/	√	/	Surgery
239	Female	74	/	√	/	/
240	Female	65	/	√	/	Chemo/Radiotherapy
241	Female	81	/	√	/	/
242	Female	76	/	√	/	Surgery
243	Female	48	/		/	Surgery
244	Female	76	/	√	/	Surgery
245	Female	65	/	√	/	Chemo/Radiotherapy
246	Female	67	/	√	/	Surgery
247	Female	83	/	√	/	Surgery
248	Female	68	/	√	/	/
249	Female	79	/	√	/	Surgery

250	Female	71	/	√	/		Surgery

/: Unkown or lost to follow up   DES: Diethylstilbestrol
